# Diagnostic Values of the QuantiFERON-TB Gold In-Tube Assay Carried out in China for Diagnosing Pulmonary Tuberculosis

**DOI:** 10.1371/journal.pone.0121021

**Published:** 2015-04-13

**Authors:** Hui Xia, Xiaomen Wang, Fabin Li, Christophe Longuet, Guy Vernet, Delia Goletti, Yanlin Zhao, Philippe H. Lagrange

**Affiliations:** 1 National Tuberculosis Reference Laboratory, Chinese Center for Disease Control and Prevention, Beijing, People’s Republic of China; 2 Tuberculosis Control Center of Heilongjiang Province, Harbin, Heilongjiang, People’s Republic of China; 3 Zhejiang Provincial Center for Disease Control and Prevention, Binjiang District, Hangzhou, Zhejiang, People’s Republic of China; 4 Fondation Mérieux, Lyon, France; 5 Department of Epidemiology and Preclinical Research, L. Spallanzani National Institute for Infectious Diseases (INMI), Rome, Italy; 6 Service de Microbiologie, Hôpital Saint Louis, Assistance Publique-Hôpitaux de Paris, Paris VII Denis Diderot University, Paris, France; Hopital Raymond Poincare - Universite Versailles St. Quentin, FRANCE

## Abstract

**Background:**

Interferon-release assays (IGRAs) for diagnosing active pulmonary tuberculosis (PTB) are not yet fully validated, particularly in high TB-endemic areas as the People's Republic of China (PRC). The aim of this report was to assess the performance of the QuantiFERON-TB Gold In-tube (QFT-GIT) and tuberculin skin test (TST), in addition to microbiological results, as contributors for diagnosing active PTB in the PRC.

**Methods/Principal Findings:**

A total of 300 PTB patients, 41 disease controls (DC) and 59 healthy community controls (HCC) were included prospectively between May 2010 and April 2011 from two provinces of the PRC (Heilongjiang and Zhejiang). The QFT-GIT and TST yielded an overall sensitivity for active TB of 80.9% and 86.2%, and a specificity of 36.6% and 26.8%, respectively. The province of origin and smear microscopy status did not significantly impact the diagnostic values for PTB. However, using the TST with a 10 mm cut-off point, a significantly higher proportion of LTBI was observed in the DC than the HCC (p=0.01). Discordant results between the QFT-GIT and TST were found among 1/3 of the PTB, HCC and DC. Two-thirds of the individuals presented TST-positive/QFT-GIT-negative discordant results. The TST-negative/QFT-GIT-positive result was not associated with age or bacillary load. Cumulative QFT-GIT and TST positive results increased the overall sensitivity (95.9%), but it was associated with a dramatic decrease of the overall specificity (24.8%) leading to a suboptimal PPV (80.1%) and a low NPV (61.1%).

**Conclusions/Significance:**

The usefulness of the QFT-GIT to diagnose active TB in high TB-endemic countries remains doubtful because like the TST, the QFT-GIT cannot distinguish between LTBI and active TB. Used as single stand-alone tests, both the QFT-GIT and TST have very limited roles in the diagnosis of active PTB. However, the combined use of SM, the TST and QFT-GIT may allow for the exclusion of ATB.

## Introduction

Tuberculosis (TB) remains a major global health problem. It ranks as the second leading cause of death from an infectious disease worldwide, after human immunodeficiency virus (HIV). In 2013, there were an estimated 9 million new cases of TB (12% HIV co-infected); 1.5 million people died from TB, including almost one million deaths among HIV-negative individuals and 360,000 among HIV-infected people [1]. Geographically, the burden of TB is highest in Asia and Africa. India and China together account for almost 35% of the world’s TB cases.

In 2013, there were an estimated 1 (0.9–1.1) million active TB (ATB) cases in China with 32.5% microbiologically confirmed TB among the total of 847,176 new pulmonary cases [1]. Consequently, around half a million new cases have been treated, and diagnosed only by clinical symptoms and abnormal chest X-rays findings without any microbiological confirmation. Chinese national guidelines have recommended obtaining three sputum specimens from patients with suspected TB [2]. This re-emphasizes the need of new biomarkers for early diagnosis of active TB [3]. Several ongoing studies in the PRC are being performed to evaluate the gold standard specimen culture and relatively new molecular diagnostic tools such as the Cepheid Xpert MTB/RIF assay [4]

In adult ATB, old and new immunological tests, such as the one-century-old tuberculin skin test (TST) and any of the new commercially available *in vitro* Interferon-gamma release assays (IGRAs), the QuantiFERON-TB (QFT-GIT) or the T SPOT-TB, are almost diagnostic adjuncts [3]. For more than one century, the TST was the reference test for diagnosing latent TB infection (LTBI) and was not commonly used for diagnosing ATB in adults. The sensitivity of both the IGRA and TST has been assessed in ATB as surrogate markers because there is no gold standard for LTBI. The accuracy of the T-SPOT-TB and QFT-GIT, in comparison to the TST, for diagnosing ATB was assessed in a systematic review and meta-analysis of published literature from both low and high TB-endemic areas [5]. Although the diagnostic sensitivity of both IGRAs was higher than that of the TST, it was still not high enough to use these assays as a rule-in test for ATB. A second systematic review and meta-analysis of published literature has been carried out to compare the respective performances of the 2 commercial IGRAs and TST in adults from low- and middle-income countries [6]. There was no consistent evidence that either of the IGRAs was more sensitive than the TST for diagnosing ATB. In low- and middle-income countries, neither the TST nor the IGRAs have any value for ruling in ATB in adults, especially in the context of HIV co-infection. On the other hand, as shown recently, a high sensitivity value of QFT-GIT and its high negative predictive value suggest a supplementary role for this test in the diagnostic exclusion of ATB, but negative QFT-GIT results should not be used alone to exclude ATB [7]. Additionally, the QFT-GIT test has limited usefulness in differentiating PTB from *Non*-Tuberculous Mycobacterial lung disease in areas with a high prevalence of LTBI [8].

Few publications have evaluated the diagnostic values of the commercially available IGRAs for PTB diagnosis in non-immunocompromised adults in the PRC: three used the T-SPOT-TB [9–11], one the QFT-G [12] and one the QFT-GIT [13]. One meta-analysis has been performed reporting the results of the homemade and commercially available IGRAs tested in China [14].

The aim of this report was to assess the performance of the QFT-GIT and TST, in addition to microbiological results, as contributors for diagnosing active PTB in two provinces of the PRC. The specificity of both tests was assessed by either evaluating individuals with a pulmonary non-TB disease or healthy community individuals.

## Materials and Methods

### Study participants

This study was approved by the Scientific Advisory Committee and Institutional Ethical Committee of the Beijing Chest Hospital. The recruitment was done between May 2010 and April 2011 in both the northeast Heilongjiang Province and southeast Zhejiang Province. Before enrolment, all the subjects were verbally informed about the study procedure and they subsequently signed the written informed consent forms, which are stored at each site. Eligible PTB patients were individuals with clinical TB symptoms; a chest X-ray was performed after each clinical examination. At the county laboratory level, a Ziehl-Neelsen (ZN) stain of the smear microscopy (SM) was carried out on 3 sputum specimens (one spot sputum, one night sputum and one morning sputum) obtained from each individual with suspected TB. This was the only microbiological test done; for this study no solid or liquid culture was carried out [15]. Individuals with a previous history of TB, those who had undergone a TST in the previous 16 months, or had silicosis, end stage renal disease, leukaemia/lymphoma or were undergoing immunosuppressive therapy were excluded from the study.

Eligible subjects consenting to the study were included into one of the following two subgroups: PTB patients and non-PTB patients, all recruited at several TB dispensaries of the Heilongjiang and Zhejiang Provinces. The first group included suspected PTB patients stratified by SM status: the SM-positive microbiologically confirmed TB patients, the SM-negative considered as “Clinical TB” patients with TB clinical symptoms and chest X-rays consistent with ATB who did not respond to 10 days of broad-spectrum antibiotics but who did respond to anti-TB treatment during the 6 month follow-up. The second group included non-PTB patients classified into 2 subgroups: one consisting of healthy community controls (HCC) and the other involving individuals with a defined pulmonary disease (Disease controls—DC) free of TB symptoms. All controls have declared no close family contact with a known PTB case. Since our settings are highly TB endemic [1], to rule out the suspicion of PTB, all the HCC were asked to give three sputum samples, have normal chest X-rays and be SM-negative. The 41 DC patients were also SM-negative and consisted of 11 bacterial pneumonia, 9 chronic bronchitis, 9 acute bronchitis, 4 asthma, 4 bronchectasia and 4 pneumonoconiosis.

The venous blood of all included individuals was collected to perform the QFT-GIT and then the TST.

### QuantiFERON-TB Gold In-tube (QFT-GIT)

QFT-GIT was used as indicated by the manufacturers (QIAGEN GmbH, Hilden, Germany). Two estimates of the QFT-GIT-positive rate were calculated: the first estimate corresponded to the calculated “clinical performance” of a biological test (the number of positive tests/total number of patients tested) including the indeterminate results as negative. The second estimate corresponded to the calculated “laboratory performance” of a biological test (the number of positive tests/number of interpretable results obtained) excluding the indeterminate results. No QFT-GIT results were communicated to the physician in charge of the patients before the end of the study, and the laboratory technicians performing the QFT-GIT were not informed of the medical status of any individual tested.

### TST

The TST was tested on the patients’ volar surface of a forearm, by intradermal injection of 5 tuberculin units (TU) of PPD-S. The size of the induration diameter was read at 72 h by a dedicated nurse. The transverse diameter induration size of the individual TST (in mm) was recorded and the qualitative interpretation of the TST was done, as per China’s guidelines for TB control [15]. The TST response was considered positive for a TST induration ≥5 mm [15].

### Statistical analysis

Median and interquartile (IQR) ranges were calculated. Diagnostic values of each test (sensitivity, specificity, positive and negative predictive value, likelihood ratio for positive and negative tests) were calculated as recommended [16]. For continuous variables, the Mann-Whitney U test was used. For categorical variables, Chi square was used. P values were considered significant if p≤0.05. The Kruskal-Wallis test was carried out to calculate the differences of IFN-γ and TST levels between the groups.

The comparison between the QFT-GIT and TST was made by using Mc Nemar Chi square tests, treating the data as paired. Agreement between the two tests was estimated using unweighted Cohen's kappa (ĸ) statistics with agreement considered ‘‘slight” for k ≤0.20, ‘‘fair” for 0.2<k≤0.40, ‘‘moderate” for 0.40<k≤0.60, ‘‘substantial” for 0.60<k≤0.80 and ‘‘optimal” for 0.80<k≤1.00. Data were analyzed using GraphPad Prism version 5.00 for Windows (GraphPad Software, San Diego, California, USA), and the GraphPad software available on their website (www.graphpad.com/quickcals.cfm).

## Results

### Populations studied

A total of 400 individuals were enrolled during the one year study period (May 2010-April 2011): 300 PTB patients and 100 controls. HIV status was unknown for all the subjects (except one HIV-infected in PTB group): their demographic characteristics are in Table 1. The proportion of males was significantly higher in the PTB patients (72.7%) than in the controls (58%) (*p*<0.0001), but it was not significant among the PTB patients (*p* = 0.51) and controls (*p* = 0.31) stratified by province of enrolment. The median age (IQR) was significantly higher in the PTB group (42 years: 28–58) than in the whole control group (35 years: 24–53) (*p*<0.0001) and among the 50 HCC enrolled in Heilongjiang (27.5 years: 20.0–40.0) (*p* = 0.0004). Among the controls in Zhejiang, the median age (IQR) of the DC patients (45 years; 32–58) was higher than the HCC (39 years; 29–51), but the difference was not significant (*p* = 0.33).

**Table 1:** Demographic and baseline parameters of the 100 controls and 300 active PTB patients stratified by province of enrolment.

Category	Controls					Active PTB patients				*p all Controls vs*. *all ATB patients*
Province	Heilongjiang	Zhejiang		*p Heilongjiang vs*. *Zhejiang*	Heilongjiang	Zhejiang		*p Heilongjiang vs*. *Zhejiang*	Total	
	Healthy donors	Healthy donors	Non-PTB patients	Total						
**Number of subjects**	50	9	41	100		150	150		300	
**Sex, N (%)**										
Male	32 (64.0)	5 (55.6)	19 (46.3)	58 (58.0)	*0*.*31*	106 (70.7)	112 (74.7)	*0*.*51*	218 (72.7)	*<0*.*0001*
Female	18 (36.0)	4 (44.4)	22 (53.7)	42 (42.0)		44 (29.3)	38 (25.3)		82 (27.3)	
**Age**										
**Median in years(IQR)**	27.5 (20–40)	39.0 (29–51)	45.0 (32–58)	35.0 (24–53)	*0*.*0004*	43.0(18–84)	41. (15–80)	*0*.*45*	42 (28–58)	*<0*.*0001*
**HIV status**										
Unknown	50	9	41	100		150	149		299	
Positive	0	0	0	0		0	1		1	
**Smear microscopy, N (%)**										
Negative	50 (100)	9 (100)	41 (100)	100 (100)		71 (47.3)	100 (66.7)		171 (57.0)	
Positive	0 (0)	0 (0)	0 (0)	0 (0)		79 (52.7)	50 (33.3)	*0*.*0011*	129 (43.0)	
**Smear grade, N / SM+ (%)**										
- (P1+)	0/50 (0)	0/9 (0)	0/41 (0)	0/100 (0)		20/79 (25.3)	28/50 (56.0)	*0*.*0007*	48/129 (37.2)	
- (P2+)	0/50 (0)	0/9 (0)	0/41 (0)	0/100 (0)		16/79 (20.3)	7/50 (14.0)	*0*.*48*	23/129 (17.8)	
- (P3+)	0/50 (0)	0/9 (0)	0/41 (0)	0/100 (0)		28/79 (35.4)	7/50 (14.0)	*0*.*0084*	35/129 (27.1)	

**Footnotes**: PTB: pulmonary tuberculosis; Non-PTB: patients with a pulmonary disease other than tuberculosis N: number of subjects; %: percentage; IQR: interquartile 25%-75%; HIV: human immunodeficiency virus; N/SM+: number of subjects in the following category among the smear-positive; P: positive (1+ to 4+).

As for inclusion criteria, all the control individuals were SM-negative. Among the PTB patients, the SM was positive in 129/300 that yielded an overall SM sensitivity of 43.0% (95% CI: 37.3–48.8) and an overall SM specificity of 100% (95% CI: 96.8–100). However, the detection rate of active TB was significantly different in PTB patients from the 2 provinces: being significantly higher in Heilongjiang (52.7%) than in Zhejiang (33.3%) (*p* = 0.0011). Additionally, the SM grade was significantly higher among those in Heilongjiang than in Zhejiang (*p* = 0.0012).

### QFT-GIT

Two samples were scored QFT-GIT indeterminate (0.5%) and were from PTB patients (0.67%; 95%CI: 0.1–2.3%); one of these 2 patients was HIV-infected.

Among the 300 PTB patients tested, the QFT-GIT showed an overall “clinical” sensitivity (indeterminate results included as negative) of 80.3%. The “laboratory” sensitivity of QFT-GIT for active TB, after exclusion of indeterminate results, was 80.9% (Table 2). The difference between the “clinical” and “laboratory” sensitivity was not significant (*p* = 0.91). Hereafter, we used only the “laboratory” sensitivity evaluating the diagnostic values of this assay.

**Table 2:** Sensitivity of the QFT-GIT and TST, using different cut-off points, in the groups of PTB patients stratified by province of enrolment.

Category	Test	N1	IR	NR	N2	Positive	Negative	% Sensitivity (95%CI)	*p* QFT-GIT vs. TST	*p* TST_5_ vs. TST_10_ orTST_15_
Heilongjiang										
	**QFT-GIT**	150	0		150	113	37	**75.3** (67.6–82.0)		
	**TST 5 mm**	150		17	133	96	37	**72.2** (63.8–79.6)	*0*.*58*	
	**TST 10 mm**	150		17	133	79	54	**59.4** (50.5–67.8)	*0*.*005*	*0*.*038*
	**TST 15 mm**	150		17	133	34	99	**25.6** (18.4–33.9)	*<0*.*0001*	*<0*.*0001*
**Zhejiang**										
	**QFT-GIT**	150	2		148	128	20	**86.5** (79.9–91.6)		
	**TST 5 mm**	150		0	150	148	2	**98.7** (95.3–99.8)	*<0*.*0001*	
	**TST 10 mm**	150		0	150	146	4	**97.3** (93.3–99.2)	*0*.*0005*	*0*.*68*
	**TST 15 mm**	150		0	150	134	16	**89.3** (83.3–93.8)	*0*.*48*	*0*.*0009*
**All PTB patients**										
	**QFT-GIT**	300	2		298	241	57	**80.9** (75.9–85.2)		
	**TST 5 mm**	300		17	283	244	39	**86.2** (81.7–90.0)	*0*.*09*	
	**TST 10 mm**	300		17	283	225	58	**79.5** (74.3–84.4)	*0*.*75*	*0*.*044*
	**TST 15 mm**	300		17	283	168	115	**59.4** (53.4–65.1)	*<0*.*0001*	*<0*.*0001*

**Footnotes**: N1: number of subjects tested; IR: indeterminate results; NR: no return visit for a TST reading; N2: number of subjects with interpretable results; % Sensitivity: percentage of sensitivity, calculated with available QFT-GIT and TST results; 95%CI: Confidence interval. QFT-GIT: QuantiFERON-TB G In-tube; TST: tuberculin skin test; PTB: pulmonary tuberculosis.

The QFT-GIT sensitivity was higher in the PTB patients in Zhejiang (86.5%) than in those in Heilongjiang (75.3%), but the difference was not significant (*p* = 0.065). The QFT-GIT results were then compared head-to-head with the TST results.

### TST

Valid TST results were available among 100% of the controls, but less in PTB patients (283/300; 94.3%) with a significantly higher proportion of non-return visits among the PTB patients in Heilongjiang (11.3%; 95% CI: 7.1–17.5) than in Zhejiang (0%; 95% CI: 0.0–2.6) (*p*<0.0001).

**Fig 1:**
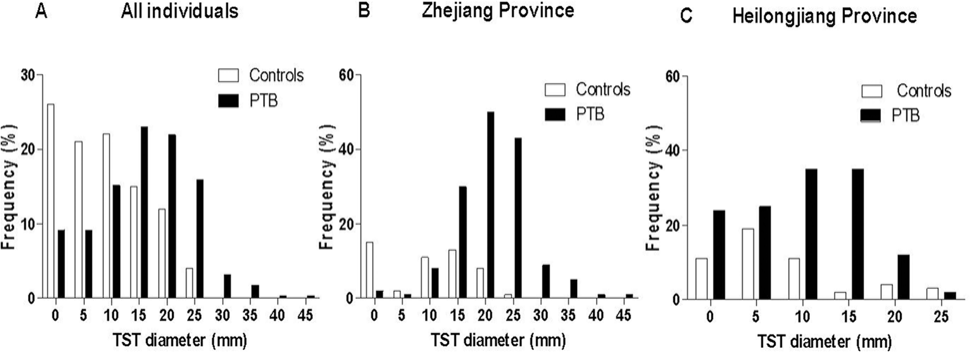
Distribution of the tuberculin skin test results in the pulmonary tuberculosis patients and controls. Histogram of the continuous distribution of the tuberculin skin test results in the pulmonary tuberculosis patients (black bar) and controls (white bar): (A) in all included individuals, (B) in those recruited in Zhejiang Province, (C) in those in Heilongjiang Province. **Abbreviations:** TST: tuberculin skin test; PTB: pulmonary tuberculosis.

The distribution of continuous TST diameter was evaluated among the PTB patients and controls stratified by province of enrolment (Fig 1A). The distribution was clearly bimodal among both the PTB patients and the controls in Zhejiang with very few individuals having a TST diameter between 1 to 10 mm (Fig 1B). In both groups, the distribution showed a first peak centered at 0 mm, followed by a second peak centered between 10 to 15 mm in the controls, and between 20 mm to 25 mm in the PTB patients. In both groups, a clear antimode at 5 mm was observed. In contrast, among both the PTB patients and controls in Heilongjiang, a higher proportion of results were less than 10 mm (up to 20%), and no clear separation of positive and negative was observed (Fig 1C). Only one single peak was observed in each group: at 5 mm for the controls and between 10 to 15 mm in the PTB patients. In such a population, the specificity of a test is highly dependent on the criterion used to score a positive result. Three cut-off points have been used to assess the diagnostic value of the TST in comparison with QFT-GIT results.

Of the 283 results available, the TST with a cut-off point of 5 mm, as recommended in the PRC, was positive in 244 PTB patients and hence yielded an overall sensitivity of 86.2% (Table 2), being significantly higher in Zhejiang (98.7%) than in Heilongjiang (72.2%)(*p* <0.0001).

Using a 10 mm cut-off point significantly reduced the overall TST sensitivity (from 86.2% to 79.5%) (*p* = 0.044). Worthy of note is the highly significant decrease of sensitivity among the PTB patients in Heilongjiang (from 72.2% to 59.4%) (*p* = 0.038), which was not the case in Zhejiang (from 98.7% to 97.3%) (*p* = 0.68).

Using a 15 mm cut-off point drastically and significantly reduced (*p*<0.0001) the overall TST sensitivity (from 86.2% to 59.4%). This was principally related to the significantly lower sensitivity in the PTB patients in Heilongjiang (72.2% to 25.6%) (*p*<0.0001), compared to those in Zhejiang (98.7% to 89.3%) (*p* = 0.48).

### Comparison between QFT-GIT and TST sensitivity for PTB: overall and stratified by province of enrolment using varying TST cut-off points

As shown in Table 2, the overall sensitivity of the QFT-GIT (80.9%) was not significantly different than those of the TST using a 5 mm or 10 mm cut-off point (p = 0.094 and *p* = 0.754, respectively). However, with a cut-off point at 15 mm, the TST sensitivity was significantly lower than the QFT-GIT sensitivity (*p*<0.0001).

Among the PTB patients in Heilongjiang, the sensitivity of the TST with a cut-off point of 5 mm was 72.2%, and was not significantly different than those of the QFT-GIT sensitivity (75.3%) (*p* = 0.588). However, with a cut-off point of 10 mm, the TST sensitivity (59.4%) decreased significantly, becoming significantly lower than those of the QFT-GIT (*p* = 0.005). Furthermore, with a 15 mm cut-off point, a profound decrease of TST sensitivity was observed, showing a highly significant difference compared to QFT-GIT sensitivity (*p*<0.0001).

In contrast, among the PTB patients in Zhejiang, the TST sensitivity, using a cut-off point at 5 mm or at 10 mm, was significantly higher compared to the QFT-GIT sensitivity (*p*<0.0001 and p = 0.005, respectively). However, with a 15 mm cut-off point, the TST sensitivity decreased significantly to 89.3% (*p* = 0.0009), now very close to the QFT-GIT sensitivity, with no significant difference (*p* = 0.48).

### QFT-GIT and TST sensitivity for LTBI diagnosis

As described above in Table 1, the control population contained a mixed population that differed according to the province of enrolment: those in Heilongjiang were exclusively HCC, whereas those in Zhejiang included 9 HCC and 41 DC patients with pulmonary disease other than tuberculosis. The sensitivity of the TST and QFT-GIT was calculated in these 3 subgroups of controls and is shown in Table 3.

**Table 3:** Comparative results of the QFT-GIT and TST, using different cut-off points, in the healthy community controls (HCC) and disease controls (DC).

Category	Test	N1	N2	Positive	Negative	% Sensitivity (95% CI)	% Specificity (95% CI)	***p*** QFT-GIT vs. TST	***p*** TST_5_ vs. TST_10_ or TST_15_
**HCC (Heilongjiang)**	**QFT-GIT**	50	50	23	27	**46.0** (31.8–60.1)	**54.0** (39.3–68.2)		
	**TST 5 mm**	50	50	30	20	**60.0** (45.2–73.6)	**40.0** (26.4–54.8)	*0*.*2292*	
	**TST 10 mm**	50	50	17	33	**34.0** (21.2–48.8)	**66.0** (51.2–78.8)	*0*.*3074*	*0*.*0158*
	**TST 15 mm**	50	50	7	43	**14.0** (5.8–26.7)	**86.0** (73.3–94.2)	*0*.*0009*	*<0*.*0001*
**HCC (Zhejiang)**	**QFT-GIT**	9	9	4	5	**44.4** (13.7–78.8)	**55.6** (21.2–86.3)		
	**TST 5 mm**	9	9	5	4	**55.6** (21.2–86.3)	**44.4** (13.7–78.8)	*1*.*000*	
	**TST 10 mm**	9	9	5	4	**55.6** (21.2–86.3)	**44.4** (13.7–78.8)	*1*.*000*	*1*.*000*
	**TST 15 mm**	9	9	3	6	**33.3** (7.5–70.1)	**66.7** (29.9–92.5)	*1*.*000*	*0*.*6372*
**All HCC**	**QFT-GIT**	59	59	27	32	**45.8** (32.7–59.3)	**54.2** (40.8–67.3)		
	**TST 5 mm**	59	59	35	24	**59.3** (45.8–71.9)	**40.7** (28.1–54.3)	*0*.*1967*	
	**TST 10 mm**	59	59	22	37	**37.3** (25.0–50.1)	**62.7** (49.1–75.0)	*0*.*4551*	*0*.*0266*
	**TST15 mm**	59	59	10	49	**16.9** (8.5–29.0)	**83.1** (71.0–91.6)	*0*.*0013*	*<0*.*0001*
**DC (Zhejiang)**	**QFT-GIT**	41	41	26	15	**63.4** (46.9–77.9)	**36.6** (22.1–53.1)		
	**TST 5 mm**	41	41	30	11	**73.2** (57.1–85.8)	**26.8** (14.2–42.9)	*0*.*4769*	
	**TST 10 mm**	41	41	26	15	**63.4** (46.9–77.9)	**36.6** (22.1–53.1)	*1*.*000*	*0*.*4769*
	**TST 15 mm**	41	41	16	25	**39.0** (24.2–55.5)	**61.0** (44.5–5.8)	*0*.*0461*	*0*.*0035*

**Footnotes**: N1: number of subjects tested; N2: number of subjects with interpretable results; % Sensitivity: percentage of sensitivity; % Specificity: percentage of specificity; 95% CI: Confidence interval; QFT-GIT: QuantiFERON-TB G In-tube; TST: tuberculin skin test; HCC: healthy community controls; DC: disease controls (individuals with a non-TB pulmonary disease).

The QFT-GIT sensitivity was not significantly different in the HCC in Heilongjiang (46.0%) than in those in Zhejiang (44.4%) (*p* = 1.0), whereas the QFT-GIT sensitivity was higher among the DC group in Zhejiang (63.4%), but the difference with the 2 other HHC groups (*p* = 0.14 and *p* = 0.45, respectively) was not significant. Similarly, the TST sensitivity, using a 5 mm cut-off point, was higher in the DC group in Zhejiang (73.0%) compared to the 2 HCC groups in Heilongjiang (60.0%) and Zhejiang (55.6%); the difference was only significant when the TST cut-off point was at 10 mm (*p* = 0.006) or 15 mm (*p* = 0.008). No significant difference was observed between the HCC in Heilongjiang and those in Zhejiang, no matter what cut-off point was used.

We then compared the sensitivity of QFT-GIT and TST among the DC group versus the combined HCC groups from the 2 provinces (Table 3). The QFT-GIT sensitivity was higher in the DC group (63.4%) than in the entire HHC group (45.8%), but the difference was not significant (*p* = 0.10). Similarly, the TST sensitivity, using a 5 mm cut-off point, was still higher in the DC group (73.2%) than in the whole HHC group (59.3%), but the difference was not significant (*p* = 0.20). However, the TST sensitivity was significantly higher in the DC group compared to the HCC group when the TST cut-off point was at 10 mm (63.4% vs. 37.3%, *p* = 0.0144) or at 15 mm (39.0% vs. 16.9%, *p* = 0.0199).

**Fig 2:**
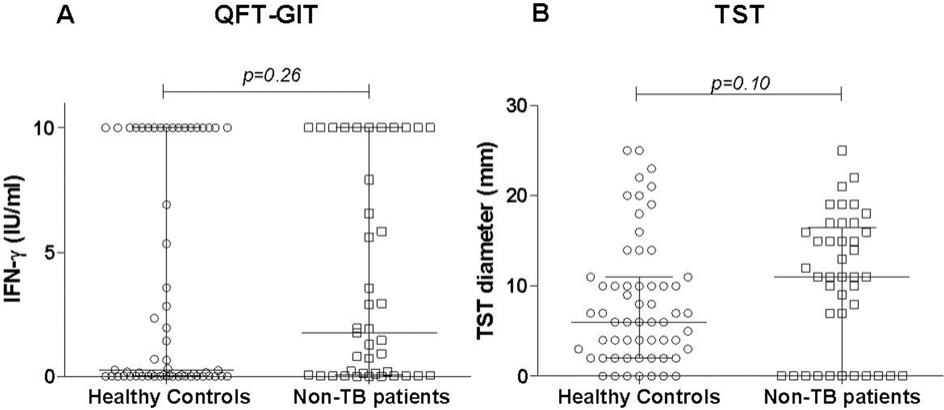
QFT-GIT and TST values in the pulmonary tuberculosis patients and controls. Individual values of the QFT-GIT (A) and TST diameter (B) in different groups of controls (healthy community controls and non-TB individuals or disease controls). Horizontal lines represent the median and interquartile range (25%-75%). **Abbreviations:** IFN: interferon-gamma, TST: tuberculin skin test; QFT-GIT: QuantiFERON TB Gold In- Tube; TB: tuberculosis.

Next, the individual quantitative QFT-GIT level (IFN-γ; IU/mL) and TST diameter were analyzed and the results are shown in Fig 2. The median (IQR) IFN-γ level was higher among the 41 DC (1.75 IU/mL: 0.05–10.0) than among the 59 HHC (0.24 IU/mL: 0.01–10.0), but the difference was not significant (*p* = 0.26). The median (IQR) TST diameter was also higher in the DC (11.0mm: 0.0–16.5) than in the HHC group (6.0mm: 2.0–11.0), but the difference was not significant (*p* = 0.10).

### Diagnostic values of QFT-IT and TST for active TB: overall and stratified by province of enrolment

The diagnostic values of the QFT-GIT and TST for active TB, with a 5 mm cut-off point, were first assessed with a specificity calculated using the negative results from the prospectively recruited DC individuals. The results are shown in Table 4. The overall positive predictive values (PPV) and negative predictive values (NPV) were not significantly different between the QFT-GIT and TST, with similar likelihood ratios (LR) for a positive and negative test. However, the positive predictive values (PPV) and negative predictive values (NPV) were higher among patients in Zhejiang than in Heilongjiang, with a significantly higher LR for a negative test when TST was considered.

**Table 4:** Diagnostic values of the QFT-GIT and TST for active tuberculosis assessed in PTB patients and disease controls.

Category	Test	% Sensitivity (95% CI)	% Specificity (95% CI)	PPV (95% CI)	NPV (95% CI)	LR1	LR2
**All PTB patients**							
	**QFT-GIT**	**80.9** (75.9–85.2)	**36.6** (22.1–53.1)	90.3 (86.1–93.5)	20.8 (12.2–32.0)	1.275	1.916
	**TST 5 mm**	**86.2** (81.7–90.0)	**26.8** (14.2–42.9)	89.1 (84.7–92.5)	22.0 (11.5–36.0)	1.178	1.942
	**TST 10 mm**	**79.5** (74.3–84.4)	**36.6** (22.1–53.1)	89.6 (85.2–93.1)	20.6 (12.0–31.6)	1.256	1.785
	**TST 15 mm**	**59.4** (53.4–65.1)	**61.0** (44.5–75.8)	91.3 (86.3–95.0)	17.9 (11.9–25.2)	1.521	1.502
**Heilongjiang**							
	**QFT-GIT**	**75.3** (67.6–82.0)	**36.6** (22.1–53.1)	81.3 (73.8–87.4)	28.7 (17.1–43.1)	1.188	1.482
	**TST 5 mm**	**72.2** (63.8–79.6)	**26.8** (14.2–42.9)	76.2 (67.8–83.3)	22.9 (12.0–37.3)	0.9865	0.982
	**TST 10 mm**	**59.4 (**50.5–67.8)	**36.6** (22.1–53.1)	75.2 (65.9–83.1)	21.7 (12.7–33.3)	0.9367	0.901
	**TST 15 mm**	**25.6** (18.4–33.9)	**61.0** (44.5–75.8)	68.0 (53.3–80.5)	20.2 (13.5–28.3)	0.6551	0.820
**Zhejiang**							
	**QFT-GIT**	**86.5** (79.9–91.6)	**36.6** (22.1–53.1)	83.1 (76.3–88.7)	42.9 (26.7–60.1)	1.364	2.711
	**TST 5 mm**	**98.7** (95.3–99.8)	**26.8** (14.2–42.9)	83.1 (76.3–88.3)	84.6 (54.6–98.1)	1.348	20.615
	**TST 10 mm**	**97.3** (93.3–99.2)	**36.6** (22.1–53.1)	84.9 (78.6–89.9)	79.0 (54.4–94.0)	1.535	13.556
	**TST 15 mm**	**89.3** (83.3–93.8)	**61.0** (44.5–75.8)	89.3 (83.3–93.8)	61.0 (44.5–75.3)	2.289	5.701

**Footnotes**: % Sensitivity: percentage of positive, calculated with available QFT-GIT and TST results; 95% CI: Confidence interval; % Specificity: percentage of negative, calculated with available QFT-GIT and TST results; PPV, positive predictive value; NPV, negative predictive value; **LR1**, likelihood ratio for a positive test; **LR2**, likelihood ratio for a negative test QFT-GIT: QuantiFERON-TB G In-tube; TST: tuberculin skin test; PTB: pulmonary tuberculosis.

Using the 2 higher TST cut-off points showed a tendency of increasing the PPV but at the expense of the NPV among all PTB patients; this was also observed among the PTB patients from each province. The best diagnostic values were observed among the patients in Zhejiang, independent of the TST cut-off point.

Then, further diagnostic values were assessed using the specificity calculated with the results from the prospectively recruited HCC individuals from both provinces; the results are shown in Table 5. The major changes were observed mostly for the NPV and LR for negative test. When the TST cut-off points increased, the PPV and LR for a positive test also increased proportionally, with a concomitant decrease of the NPV and LR for a negative test.

**Table 5:** Diagnostic values of the QFT-GIT and TST for active tuberculosis assessed in PTB patients and healthy community controls.

Category	Test	% Sensitivity (95% CI)	% Specificity (95% CI)	PPV	NPV	LR1	LR2
**All PTB patients**							
	**QFT-GIT**	**80.9** (75.9–85.2)	**54.2** (40.8–67.3)	89.9 (85.7–93.3)	36.0 (26.1–46.8)	1.767	4.972
	**TST 5 mm**	**86.2** (81.7–90.0)	**40.7** (28.1–54.3)	87.5 (83.0–91.1)	38.1 (26.2–51.2)	1.453	2.949
	**TST 10 mm**	**79.5** (74.3–84.4)	**62.7** (49.2–75.0)	91.1 (86.6–94.3)	39.0 (29.1–49.5)	2.132	3.059
	**TST 15 mm**	**59.4** (53.4–65.1)	**83.1** (71.0–91.6)	94.4 (89.9–97.3)	29.9 (23.0–37.5)	3.502	2.047
**Heilongjiang**							
	**QFT-GIT**	**75.3** (67.6–82.0)	**54.2** (40.8–67.3)	80.7 (73.2–86.9)	46.4 (34.3–58.8)	1.646	2.194
	**TST 5 mm**	**72.2** (63.8–79.6)	**40.7** (28.1–54.3)	73.3 (64.9–80.6)	39.3 (27.0–52.7)	1.217	1.464
	**TST 10 mm**	**59.4 (**50.5–67.8)	**62.7** (49.2–75.0)	78.2 (68.9–85.8)	40.7 (30.5–51.5)	1.593	1.544
	**TST 15 mm**	**25.6** (18.4–33.9)	**83.1** (71.0–91.6)	77.3 (62.2–88.5)	33.1 (25.6–41.3)	1.508	1.117
**Zhejiang**							
	**QFT-GIT**	**86.5** (79.9–91.6)	**54.2** (40.8–67.3)	82.6 (75.7–88.2)	61.5 (47.0–74.7)	1.890	4.015
	**TST 5 mm**	**98.7** (95.3–99.8)	**40.7** (28.1–54.3)	80.9 (74.4–86.3)	92.3 (74.9–99.1)	1.663	15.074
	**TST 10 mm**	**97.3** (93.3–99.2)	**62.7** (49.2–75.0)	86.9 (80.9–91.6)	90.2 (76.9–97.3)	2.610	23.222
	**TST 15 mm**	**89.3** (83.3–93.8)	**83.1** (71.0–91.6)	93.1 (87.6–96.6)	75.4 (63.1–85.2)	5.271	7.766

**Footnotes**: % Sensitivity: percentage of positive, calculated with available QFT-GIT and TST results; 95% CI: Confidence interval; % Specificity: percentage of negative, calculated with available QFT-GIT and TST results; PPV, positive predictive value; NPV, negative predictive value; **LR1**, likelihood ratio for a positive test; **LR2**, likelihood ratio for a negative test QFT-GIT: QuantiFERON-TB G In-tube; TST: tuberculin skin test; PTB: pulmonary tuberculosis.

### Agreement and concordance between the QFT-GIT and TST

The overall agreement assessed in the entire group of 381, which included individuals with concurrently available results of the QFT-GIT and TST using the 5 mm cut-off point (Table 6), was very high (71.9%) with only a fair concordance (κ = 0.212). The 107 discordant results comprised a significantly higher proportion of subjects who were TST-positive but QFT-GIT-negative (TST+/QFT-) (61.7%), compared to those who were TST-negative but QFT-GIT-positive (TST-/QFT+) (38.3%) (p = 0.001).

**Table 6:** Agreement and concordance value (Kappa coefficient) of the QFT-GIT and tuberculin skin test, using different cut-off points.

	TST	QFT-GIT Number (percentage)					
		All	Negative	Positive	Agreement (%)	Kappa coefficient	***p*** *
All individuels**							
	< 5 mm	75 (100)	**34** (45.3)	**41** (54.7)			
	≥ 5 mm	306 (100)	**66** (21.6)	**240** (78.4)	71.9	0.212	<0.0001
	< 10 mm	110 (100)	**47** (42.7)	**63** (57.3)			
	≥10 mm	271 (100)	**53** (19.6)	**218** (80.4)	69.6	0.238	<0.0001
	< 15 mm	193 (100)	**67** (34.7)	**126** (65.3)			
	≥ 15 mm	188 (100)	**33** (17.6)	**155** (82.4)	58.3	0.170	0.0002
PTB**							
	< 5 mm	39 (100)	**10** (25.6)	**29** (75.4)			
	≥ 5 mm	242 (100)	**43** (17.8)	**199** (82.2)	74.4	0.068	0.2703
	< 10 mm	58 (100)	**17** (29.3)	**41** (70.7)			
	≥10 mm	223 (100)	**36** (16.1)	**187** (83.9)	72.6	0.136	0.0366
	< 15 mm	115 (100)	**28** (24.3)	**87** (75.7)			
	≥ 15 mm	166 (100)	**25** (16.1)	**141** (84.9)	60.1	0.101	0.0625
**Controls**							
	< 5 mm	36 (100)	**24** (66.7)	**12** (33.3)			
	≥ 5 mm	64 (100)	**23** (35.9)	**41** (64.1)	65.0	0.288	0.0037
	< 10 mm	52 (100)	**30** (57.7)	**22** (42.3)			
	≥10 mm	48 (100)	**17** (35.4)	**31** (64.6)	61.0	0.221	0.0293
	< 15 mm	74 (100)	**38** (51.4)	**36** (48.4)			
	≥ 15 mm	26 (100)	**9** (34.6)	**17** (65.4)	55.0	0.125	0.1736

**Footnotes**: QFT-GIT: QuantiFERON-TB G in tube; TST: tuberculin skin test; PTB: pulmonary tuberculosis; Percentages quoted in () are percentages by rows.

* Fisher’s exact test.

**: 19 unavailable results (17 patients did not return for TST readings and 2 patients had indeterminate QFT-GIT results).

The results were further analyzed in groups stratified by their TB status (Table 6). The agreement between the 2 tests was not significantly higher in the PTB group (74.4%) than in the entire group of controls (65.0%) (*p* = 0.07). To note: among the PTB, the concordance was slight (ĸ = 0.068), whereas a fair concordance was obtained in the entire group of controls (ĸ = 0.288). The proportion of discordant results was similar in both groups and involved a significantly higher proportion of subjects who were TST+/QFT- compared to the subjects who were TST-/QFT+ in both groups (PTB: *p* = 0.0299; controls: *p* = 0.0162).

The concordance was further evaluated when the TST cut-off point rose from 5 mm to 10 mm and 15 mm (Table 6). No significant change of the agreement was observed with a 10 mm cut-off point compared to the results obtained with the 5 mm cut-off point among each group evaluated. To note: there was an increase of the overall k coefficient, mostly marked in the PTB. Using a 15 mm cut-off point significantly decreased the agreement in the PTB (from 74.4% to 60.1%; *p* = 0.004) and in all the included subjects (71.9% to 58.3%: *p* = 0.001). No significant change was observed in the control group using the different cut-off points.

The impact of the province of recruitment upon the agreement and the concordance between the 2 tests was further analyzed among the PTB patients and the results are shown in Table 7. The agreement was significantly lower in patients from Heilongjiang (63.2%) compared to those from Zhejiang (86.5%)(*p*<0.0001), with a slight kappa coefficient in both groups. A significantly higher proportion of TST+/QFT- patients was observed among those from Zhejiang (100%) compared to those from Heilongjiang (46.0%) (*p* = 0.0015). By contrast, the agreement and concordance were similar between the HCC and DC groups (*p* = 0.83) (Table 7). Similar proportions of TST+/QFT- and TST-/QFT+ were observed in both control groups.

**Table 7:** Agreement and concordance value (Kappa coefficient) of the QFT-GIT and TST results in the PTB patients stratified by province of enrolment, and in the 2 subgroups (HCC and DC) of controls.

		TST					
PTB patients	QFT-GIT	≥ 5 mm	< 5 mm	Total	Agreement (%)	Κ coefficient	***p*** *
**Heilongjiang**	**Positive**	**73**	**27**	**100**			
	**Negative**	**23**	**10**	**33**	62.4	0.031	*0*.*8231*
	Total**	**96**	**37**	133**			
**Zhejiang**	**Positive**	**126**	**0**	**126**			
	**Negative**	**20**	**2**	**22**	86.5	0.145	*0*.*0212*
	Total***	**146**	**2**	148***			
**Controls**							
**HCC**	**Positive**	**20**	**7**	**27**			
	**Negative**	**15**	**17**	**32**	62.7	0.265	*0*.*0616*
	**Total**	**35**	**24**	**59**			
**DC**	**Positive**	**21**	**5**	**6**			
	**Negative**	**9**	**6**	**15**	65.9	0.220	*0*.*2720*
	**Total**	**30**	**11**	**41**			

**Footnotes**: QFT-GIT: QuantiFERON-TB G In-tube; TST: tuberculin skin test; a 5 mm cut-off point was used for the TST; PTB: pulmonary tuberculosis; HCC: Healthy Community controls; DC: Disease controls.

* Fisher’s exact test;

**: 17 patients did not return for a TST reading;

*** 2 patients had indeterminate QFT-GIT results.

Several factors have been described to explain the discordant results between the IGRAs and TST: the bacillary load in the active TB patients, the BCG vaccination status and the age among subjects without active TB [17]. We further analyzed these factors.

### Impact of the sputum bacillary load on the sensitivity of the *QFT-GIT and TST* in PTB patients

We analyzed the qualitative (positive or negative results) and quantitative individual results of the QFT-GIT and TST in the whole group of PTB patients stratified into two subgroups: the patients with microbiological TB confirmation (SM-positive) and the second without microbiological confirmation, classified as “clinical TB” (SM-negative but with an efficient response to an anti-tuberculosis therapy of 6 months).

First, we analyzed the qualitative results. Among the PTB patients stratified by SM status (Table 8), no significant difference of QFT-GIT sensitivity was observed between the SM-positive (82.7%) and SM-negative (79.5%) patients (p = 0.55). Similarly, no significant difference of TST sensitivity, using a 5 mm cut-off point, was observed between the SM-positive (89.6%) and SM-negative (83.9%) patients (p = 0.22).Finally, no significant difference was observed between QFT-GIT and TST sensitivity in the SM-negative (*p* = 0.32) or SM-positive PTB patients (*p* = 0.14). Likewise, using a 10 mm cut-off point, the TST and QFT-GIT sensitivity was not significantly different among the SM-negative (*p* = 0.79) compared to SM-positive patients (*p* = 0.87). In contrast, due to the drastic reduction of TST sensitivity using a 15 mm cut-off, now the QFT-GIT sensitivity was significantly higher than the TST both among the SM-negative and SM-positive patients (*p*<0.0001).

**Table 8:** Sensitivity of the QFT-GIT and TST, using different cut-off points, in the groups of PTB patients stratified by sputum smear microscopy status.

Category	Test	N1	IR	NR	N2	Positive	Negative	% Sensitivity (95% CI)	*p* QFT-GIT vs. TST	*p* TST_5_ vs. TST_10_orTST_15_
**Smear-positive**	**QFT-GIT**	128	1		127	105	22	**82.7** (75.01–88.8)		
	**TST 5 mm**	128		13	115	103	12	**89.6** (82.6–94.5)	*0*.*141*	
	**TST10 mm**	128		13	115	94	21	**81.7** (73.5–88.3)	*0*.*86*	*0*.*13*
	**TST 15 mm**	128		13	115	61	54	**53.0** (43.5–62.4)	*<0*.*0001*	*<0*.*0001*
**Smear-negative**	**QFT-GIT**	172	1		171	136	35	**79.5** (72.7–85.3)		
	**TST 5 mm**	172		4	168	141	27	**83.9** (77.5–89.1)	*0*.*32*	
	**TST10 mm**	172		4	168	131	37	**78.0** (70.9–84.0)	*0*.*79*	*0*.*21*
	**TST 15 mm**	172		4	168	107	61	**63.7** (55.9–71.0)	*<0*.*0001*	*0*.*0016*

**Footnotes**: QFT-GIT: QuantiFERON-TB G In-tube; TST: tuberculin skin test; PTB: pulmonary tuberculosis; N1: number of subjects tested; IR: indeterminate results; NR: no return visit for a TST reading; N2: number of subjects with interpretable results; % Sensitivity: percentage of sensitivity, calculated with available QFT-GIT and TST results; 95% CI: Confidence interval.

**Fig 3:**
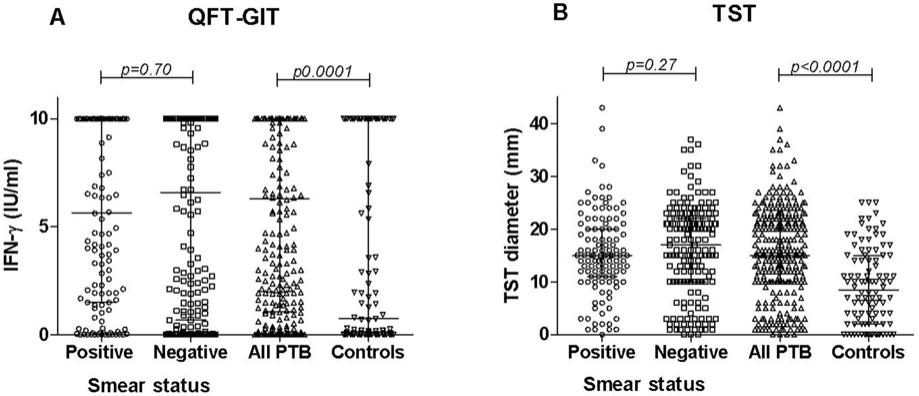
QFT-GIT and TST values in pulmonary TB patients stratified by smear microscopy status and in the group of controls. Individual values of the QFT-GIT (A) and TST diameter (B) in the groups of pulmonary TB patients stratified by smear microscopy status and in the entire group of controls. Horizontal lines represent the median and interquartile range (25%-75%). **Abbreviations:** IFN: interferon-gamma, TST: tuberculin skin test; QFT-GIT: QuantiFERON TB Gold In-Tube; PTB: pulmonary tuberculosis.

We then analyzed the quantitative results. The individual IFN-γ production (IU/ml) and TST (diameter in mm) results obtained among all enrolled individuals are presented in Fig 3. The median (IQR) IFN-γ level (Fig 3A) was significantly higher among the PTB (6.32 IU/mL; 1.01–10.0) than the whole control group (0.76 IU/mL; 0.01–10.0) (*p*<0.0001). Similarly, the median (IQR) TST diameter (Fig 3B) was significantly higher among the PTB (15.0mm; 10.0–22.0) than the whole control group (8.5 mm; 2.0–15.0) (*p*<0.0001). The median (IQR) IFN-γ level was higher among the 172 SM-negative (6.59 IU/mL; 0.68–10.0) than the 128 SM-positive (5.65 IU/mL; 1.5–10.0) PTB, but the difference was not significant (*p* = 0.70). Similarly, the median (IQR) TST diameter was higher among the SM-negative (17.0mm; 10.0–22.0) than the SM-positive (15.0mm; 11.0–20.0) PTB, but the difference was not significant (*p* = 0.27).

**Fig 4:**
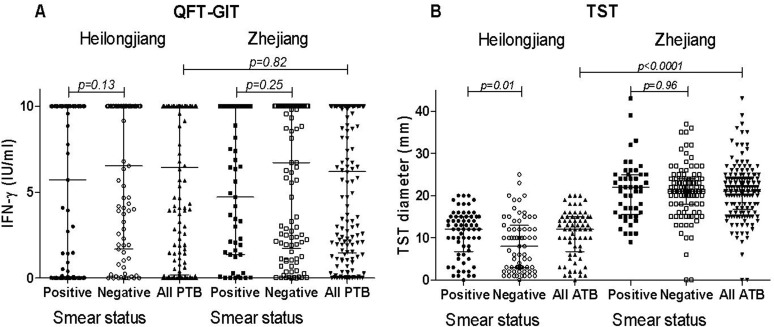
QFT-GIT and TST values in the different groups of PTB patients according to the province of recruitment and stratified by smear microscopy status. Individual values of the QFT-GIT (A) and TST diameter (B) in the different groups of PTB patients according to the province of recruitment and stratified by smear microscopy status. Horizontal lines represent the median and interquartile range (25%-75%). **Abbreviations:** IFN: interferon-gamma, TST: tuberculin skin test; QFT-GIT: QuantiFERON TB Gold In- Tube; PTB: pulmonary tuberculosis.

The impact of the smear status upon the individual IFN-γ production (IU/ml) and TST (diameter in mm) results was further analyzed among the PTB patients stratified by province of enrolment. As shown in Fig 4A, the IFN-γ level did not differ significantly between the PTB in Heilongjiang (6.43 IU/mL; 0.19–10.0) and those in Zhejiang (6.20 IU/mL; 1.47–10.0) (*p* = 0.82), and no significant impact of the smear status on the IFN-γ responses was observed among the patients from the 2 provinces (*p*>0.1). In contrast, as shown in Fig 4B, the TST diameter was significantly higher among the PTB from Zhejiang (21.0mm; 16.75–24.0) compared to those from Heilongjiang (10.0mm; 3.0–15.0) (*p*<0.0001). To note: a significantly lower TST diameter was found among the SM-negative (10.0 mm: 3.0–13.0) than among the SM-positive (12.0 mm: 6.75–15.0) PTB patients (*p* = 0.01) in Heilongjiang, but there was no significant impact of the smear status on the TST diameter among the Zhejiang PTB patients (*p* = 0.96).

### Effect of the smear microscopy grade on the QFT-GIT and TST results

The entire population of PTB patients was further stratified into 5 subgroups according to SM grade (Smear-negative and Smear-positive with one+ to four+) and we further analyzed the qualitative (positive or negative results) and quantitative values of the QFT-GIT and TST in these 5 subgroups of pulmonary TB patients.

First, we evaluated the qualitative results. As shown in Table 9, the smear grade has no influence on the QFT-GIT (*p* = 0.14) sensitivity or TST sensitivity (*p* = 0.51) using a 5 mm cut-off point.

**Table 9:** Comparison of the QFT-GIT and TST results in the groups of PTB patients stratified by sputum smear microscopy grade.

Smear grade	Negative	P (+)	P (++)	P (+++)	P (++++)	***p*** value
Number of positive / tested with interpretable results						
% Sensitivity (95% CI)						
**QFT-GIT (≥0.35 IU/mL)**	136/171^(a)^	41/47^(a)^	18/23	25/35	22/23	
	**79.5** (72.7–85.3)	**87.2** (74.3–95.2)	**78.3** (56.3–92.5)	**71.4** (53.7–85.4)	**95.7** (78.1–99.9)	*0*.*14*
**TST (≥5 mm)**	141/168^(b)^	43/47^(c)^	19/20^(d)^	26/30^(e)^	16/19^(f)^	
	**83.9** (77.5–89.1)	**91.5** (79.6–97.6)	**95.0** (75.1–99.9)	**86.7** (69.3–96.2)	**84.2** (60.4–96.6)	*0*.*51*

**Footnotes**: QFT-GIT: QuantiFERON-TB G In-tube; TST: tuberculin skin test; PTB: pulmonary tuberculosis; % Sensitivity: percentage of sensitivity, calculated with available QFT-GIT and TST results; 95%CI: Confidence interval; P: smear-positive with varying grade (+ to ++++).

one patient with indeterminate QFT-GIT results

4 patients with no return visits for TST readings

1 patient with no return visit for a TST reading

3 patients with no return visits for TST readings

5 patients with no return visits for TST readings

4 patients with no return visits for TST readings

**Fig 5:**
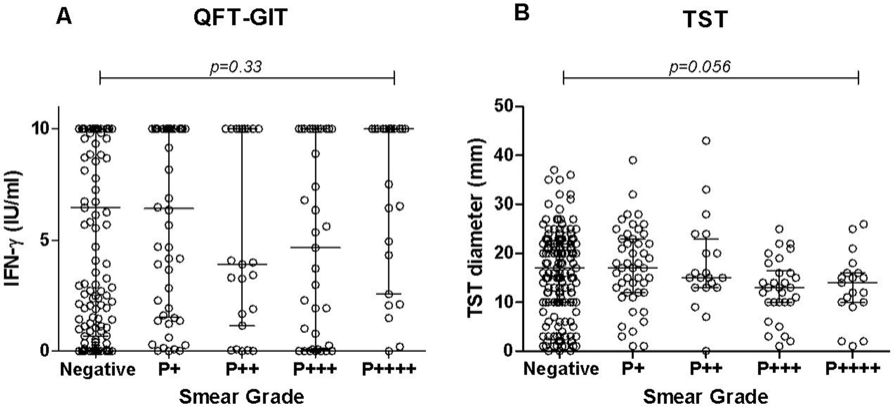
QFT-GIT and TST values in the different groups of PTB patients stratified by smear microscopy status. Individual values of the QFT-GIT (A) and TST diameter (B) in the different groups of PTB patients stratified by smear microscopy grade. Horizontal lines represent the median and interquartile range (25%-75%). **Abbreviations:** IFN: interferon-gamma, TST: tuberculin skin test; QFT-GIT: QuantiFERON TB Gold In-Tube; PTB: pulmonary tuberculosis; P: positive smear microscopy and their grade (P+ to P++++).

Then, we evaluated the quantitative results. The impact of the smear grade on the individual IFN-γ levels and TST results was further analyzed and the results are shown in Fig 5. No significant influence of the smear grade was observed on the IFN-γ level (*p* = 0.33) or on the TST results (*p* = 0.056).

### Impact of age on the sensitivity of the *QFT-GIT and TST*

The high frequency of the TST+/QFT- results has been reported to be associated with a prior BCG vaccination [17] and/or with a sensitization with Non-Tuberculous Mycobacteria (NTM) both in the PTB and non-PTB patients [18]. The high prevalence of NTM in the PRC [19] and the extremely high coverage of the BCG vaccination at birth (99%) with a recommended revaccination at 1 year of age for the TST non-converters according to Chinese regulations [20], might be the most probable factors associated with the preceding TST+/QFT- discordant results, where the cross reactions are detected only by the TST. To note that the BCG revaccination at one year of age has been cancelled in the PRC since the 1990’s. We then assessed the impact of only one BCG vaccination at birth versus the recommended two BCG vaccination schedule before 1990 among the entire control group stratified by age. The concordant and discordant results were calculated in the three subgroups as following: individuals under 16 years of age (BCG vaccinated only once at birth), those aged 16 to 60 years (receiving at least two BCG vaccinations) and those aged >60 years (no BCG vaccination) (Table 10). Among the first group (<16 years old), the agreement between the 2 assays was 100.0% with an “optimal” ĸ coefficient of 1.00. These results were significantly higher compared to those of the second (17–60 years old) group (p = 0.0119), but no significant difference was observed with the third (subjects > 60 years) group (p = 0.23).

**Table 10:** Agreement and concordance value (Kappa coefficient) of the QFT-GIT and TST results in the 100 controls stratified by age.

Age (years)	QFT-GIT	TST			Concordance (%)	Agreement (κ)
		≥ 5 mm	< 5 mm	Total		
<16*	**Positive**	6	0	6	**100.00**	**1.00**
	**Negative**	0	3	3		
	**Total**	6	3	9		
16–60**	**Positive**	28	10	38	**57.7**	**0.160**
	**Negative**	23	17	40		
	**Total**	50	28	78		
>60***	**Positive**	7	2	9	**76.9**	**0.494**
	**Negative**	1	3	4		
	**Total**	8	5	13		

**Footnotes**: QFT-GIT: QuantiFERON-TB G In-tube; TST: tuberculin skin test; a 5 mm cut-off point was used for TST.

*: one BCG vaccination at birth,

**: two BCG vaccinations (the first at birth and the second at one year if the TST was negative),

***: no BCG vaccination

### Relationship between the QFT-GIT and TST results in the PTB patients and controls from the 2 provinces

**Fig 6:**
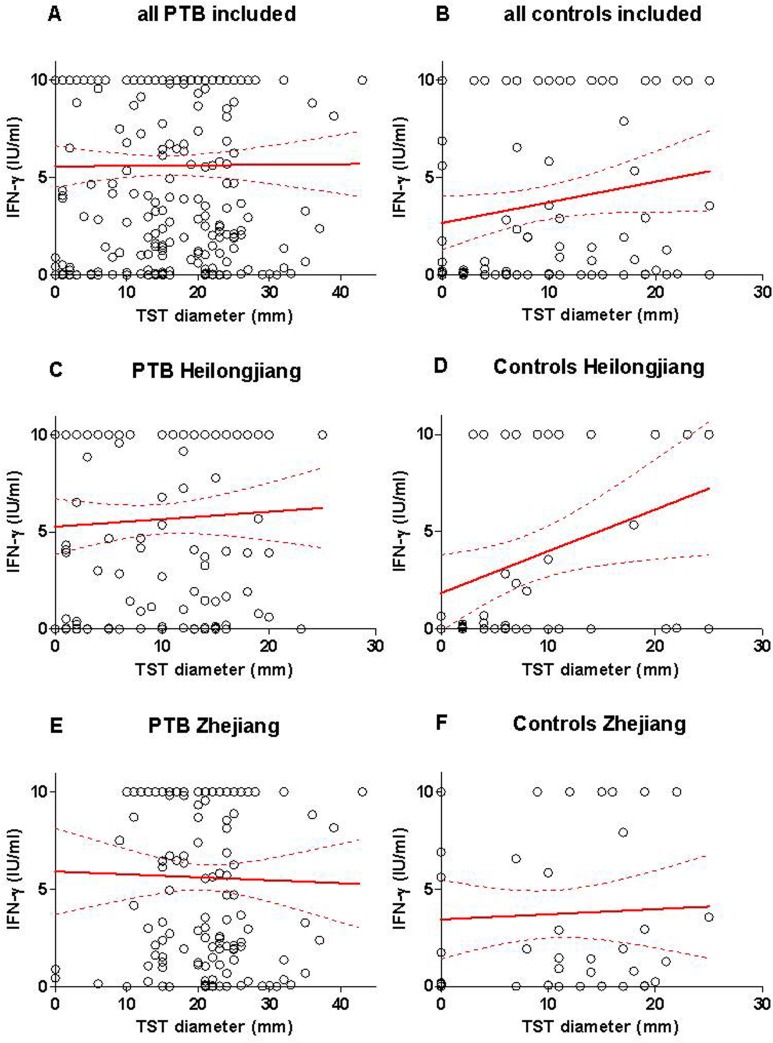
Correlation between the individual IFN-γ levels and TST diameter. Correlation between the individual IFN-γ levels (IU/ml) and TST diameter (in mm) among (A) all the active PTB patients (A), all the control individuals (B), and among those recruited in Heilongjiang Province (C and D), or recruited in Zhejiang Province (E and F). Continuous lines represent the median, and hatched lines the interquartile range (25%-75%). **Abbreviations:** PTB: pulmonary tuberculosis; IFN-γ: interferon-gamma; IU: international unit. TST: tuberculin skin test.

The relationship between the individual QFT-GIT level (IU/ml) and the TST diameter (in mm) was further analyzed among the PTB patients and control groups from each province. Among all the included PTB patients, the IFN-γ level was not significantly associated with the TST diameter (R^2^ = 0.00003: *p* = 0.93), showing a non-significant increase of the IFN-γ level (slope: +0.003±0.030) with the TST diameter (Fig 6A). Among all the included controls, the IFN-γ level was not significantly associated with the TST diameter (R^2^ = 0.0312: *p* = 0.079), showing a non-significant increase of the IFN-γ level (slope: +0.106±0.060) with the TST diameter (Fig 6B).

In the PTB in Heilongjiang, no significant association between the IFN-γ level and TST was observed (R2 = 0.002761; *p* = 0.548), showing a non-significant increase of the IFN-γ level (slope: +0.0384±0.0638) with the TST diameter (Fig 6C). In contrast among the controls in Heilongjiang, the IFN-γ level was significantly associated with the TST diameter (R^2^ = 0.0992: *p* = 0.0259), showing a significant increase of the IFN-γ level (slope: +0.2143±0.0932) with the TST diameter (Fig 6D).

Among the PTB in Zhejiang, the IFN-γ level was not significantly associated with the TST diameter (R^2^ = 0.00058: *p* = 0.77), but showed a non-significant decrease of the IFN-γ level (slope: -0.0150±0.0512) with the TST diameter (Fig 6E). Similarly, among the controls in Zhejiang, the IFN-γ level was not significantly associated with the TST diameter (R^2^ = 0.0023: *p* = 0.741), showing a non-significant increase of the IFN-γ level (slope: +0.02640±0.0793) with the TST diameter (Fig 6F).

### Combination of smear microscopy with TST and QFT-GIT

Because the results of the two immune-based tests have shown that they are not affected by the smear status (Table 8 and Fig 4) and that the IGRA and TST quantitative results were not related **(** Fig 6 **)**, we assessed whether the possible combination of the microbiological and immunological tests might improve the ultimate detection of PTB cases.

We evaluated the sensitivity and specificity for ATB diagnosis of the combined tests among the 281 PTB patients and 100 controls who were concomitantly evaluated with SM, the QFT-GIT and TST, using 3 different cut-off points (see Tables A–F in S1 File). These results indicate that in PTB, the combination of the available SM with immunological tests did increase the possibility of detecting PTB and was potentially important to rule out active TB in suspected patients with all negative tests.

Using the TST with a 5 mm cut-off point, the PPV and NPV of the cumulative QFT-GIT plus TST results among all PTB were 77.9% and 69.7%, respectively; the positive and negative LR values were 1.252 and 6.390 (Table 11). The PPV and NPV of the cumulative (QFT-GIT plus TST plus SM) results were 78.1% and 76.7%, respectively, with a positive and negative LR of 1.266 and 9.200. This high cumulative sensitivity, and at the same time, very low cumulative specificity, indicates that the association of tests could be helpful in ruling out PTB in those resulting negative for the 3 tests, but will not help rule in the PTB patients.

**Table 11:** Diagnostic values of the single test or combined test for active tuberculosis assessed in the PTB patients and healthy community controls.

Test	% Sensitivity (95% CI)	% Specificity (95% CI)	PPV	NPV	LR1	LR2
**SM**	**43.0** (37.3–48.8)	**100** (96.4–1.00)	100 (97.2–1.00)	36.9 (31.1–42.9)	NC	1.754
**QFT**	**80.9** (75.9–85.2)	**47.0** (39.6–57.2)	82.0 (77.1–86.2)	45.2 (35.4–55.3)	1.526	2.461
**SM+QFT**	**88.3** (84.1–91.7)	**47.0** (39.6–57.2)	83.2 (78.6–87.2)	57.3 (45.9–68.2)	1.665	4.017
**TST (5 mm)**	**86.2** (81.7–90.0)	**35.0** (25.7–45.2)	79.0 (74.0–83.4)	47.3 (35.6–59.3)	1.326	2.536
**SM+TST (5 mm)**	**95.1** (91.8–97.3)	**35.0** (25.7–45.2)	80.5 (75.9–84.7)	71.4 (56.7–83.4)	1.462	7.143
**QFT+TST (5 mm)**	**96.4** (93.6–98.3)	**23.0** (15.2–32.5)	77.9 (73.1–82.1)	69.7 (51.3–84.4)	1.252	**6.390**
**SM+QFT+TST (5 mm)**	**97.5** (94.9–99.0)	**23.0** (15.2–32.5)	78.1 (73.4–82.3)	76.7 (57.7–90.1)	1.266	**9.200**
**TST (10 mm)**	**79.5** (74.3–84.1)	**52.0** (41.8–62.1)	82.4 (77.4–86.7)	47.3 (38.7–57.0)	1.656	2.537
**SM+TST (10 mm)**	**86.9** (82.4–90.6)	**52.0** (41.8–62.1)	83.7 (78.9–87.7)	58.4 (47.5–68.8)	1.811	3.969
**QFT+TST (10 mm)**	**94.3** (90.9–96.7)	**30.0** (21.2–40.0)	79.1 (74.4–83.3)	65.2 (49.8–78.7)	1.347	**5.263**
**SM+QFT+TST (10 mm)**	**95.7** (92.7–97.8)	**30.0** (21.2–40.0)	79.4 (74.7–83.5)	71.4 (55.4–84.3)	1.368	**6.977**
**TST (15 mm)**	**58.3** (52.3–64.1)	**74.0** (64.3–82.3)	86.4 (80.7–90.9)	38.5 (31.0–45.8)	2.242	1.775
**SM+TST (15 mm)**	**77.4** (72.1–82.1)	**74.0** (64.3–82.3)	89.4 (84.8–93.0)	53.6 (44.9–62.2)	2.976	3.274
**QFT+TST (15 mm)**	**89.7** (85.5–93.0)	**39.0** (29.9–49.3)	80.5 (75.7–84.8)	57.4 (44.8–69.3)	1.470	**3.786**
**SM+QFT+TST (15 mm)**	**92.2** (88.4–95.0)	**39.0** (29.9–49.3)	80.9 (76.2–85.1)	63.9 (50.6–75.8)	1.511	**5.000**

**Footnotes**: PTB: pulmonary tuberculosis; **SM**: smear microscopy; **QFT**: QuantiFERON-TB G In-tube; **TST**: tuberculin skin test (cut-off point); % Sensitivity: percentage of positive, calculated with available SM, QFT-GIT and TST results; 95% CI: Confidence interval; % Specificity: percentage of negative, calculated with available SM, QFT and TST results; **PPV**: positive predictive value; NPV: negative predictive value; **LR1**, likelihood ratio for a positive test; **LR2**, likelihood ratio for a negative test.

Using the TST with a 10 mm cut-off point, the PPV and NPV of the cumulative QFT-GIT plus TST results among all PTB were 79.1% and 65.2%, respectively; the positive and negative LR values were 1.347 and 5.263 (Table 11). The PPV and NPV of the cumulative QFT-GIT with TST and SM results were 79.4% and 71.1%, respectively, with a positive and negative LR of 1.368 and 6.977.

Using the TST with a 15 mm cut-off point, the PPV and NPV of the cumulative QFT-GIT with TST results among all PTB were 80.5% and 57.4%, respectively; the positive and negative LR values were 1.470 and 3.786 (Table 11). The PPV and NPV of the cumulative QFT-GIT with TST and the SM results were 80.9% and 63.8%, respectively, with a positive and negative LR of 1.511 and 5.000.

## Discussion

### QFT-GIT results

Few publications have evaluated the diagnostic values of the commercially available IGRAs for ATB diagnosis in non-immunocompromised adults in the PRC. The first 3 publications were from Fudan University (Shanghai) and assessed the potential diagnostic values of the T-SPOT-TB [9–11]. Zhang *et al*. reported a T-SPOT-TB sensitivity of 87.6% in 89 PTB patients with a specificity of 78.9% calculated in 57 healthy blood donors. When the T-SPOT-TB and TST were concurrently tested among 38 PTB patients, the T-SPOT-TB sensitivity (94.7%) was significantly higher than that of the TST (73.7%), and the TST specificity (37.7%) was significantly lower than the T-SPOT-TB (81.1%) (p<0.0001) [10]. Feng *et al*. reported similar results of the T-SPOT-TB with an overall sensitivity of 94.7% in 75 ATB patients and a specificity of 84.1%, evaluated in 107 non-TB patients [11]; no significant difference in sensitivity was found between the microbiologically confirmed ATB (42/45: 93.3%) and the “clinical ATB” cases (29/30: 96.7%). Moreover, the sensitivity did not differ significantly in the pulmonary (43/45: 95.6%) and extrapulmonary ATB (28/30: 93.3%). However, the specificity was significantly lower in the pulmonary group (69.2%) than in the extrapulmonary group (88.9%) [11].

A more recent systematic and meta-analysis that included the results of 10 studies assessing a mix of commercially available in-house T-SPOT-TB has been carried out in China [14]. It showed a similar estimated pooled sensitivity of 88% (95% CI: 86–91) and pooled specificity of 89% (95% CI: 86–92), with a positive LR of 8.86 (95% CI: 5.42–14.46), and a diagnostic odds ratio of 88.2 (95% CI: 41.8–186.1).

The publication reported the comparison of the QFT-GIT results with those obtained with an in-house ELISPOT performed in Shenzhen [13]. When the QFT-GIT and ELISPOT were concurrently tested among 49 ATB patients and 101 healthy controls, the sensitivity and specificity of the QFT-GIT (80.9% and 73.3%) was comparable to those of the homemade ELISPOT (83.0% and 70.3%).

Here we show that the QFT-GIT sensitivity (80.6%) was higher than that reported by Liu *et al*. using the QFT-G [12], but was very close to the results obtained by Chen *et al*. using the QFT-GIT [13] and similar to that using the T-SPOT-TB in the meta-analysis [14]. However, our study showed a dramatically lower overall specificity (47.0%) of the QFT-GIT compared to the 73.3% found by Chen *et al*. [13] and the 89% of the T-SPOT-TB described in the meta-analysis [14]. Because the IGRAs cannot differentiate ATB from LTBI [21–24], such differences between our results and those described above could be related to varying numbers of LTBI cases among the controls recruited at different settings. As shown in our study, a higher proportion of QFT-GIT-positive results was observed in the control individuals in Zhejiang (60.0%) compared to those in Heilongjiang (46.0%), but the difference was not significant (p = 0.22). The variable proportion of QFT-GIT-positive results among the controls was not linked *per se* to the site of recruitment, but was associated with the heterogeneity of the control population involving HCC and DC in Zhejiang. As a matter of fact, a comparable proportion of the QFT-GIT-positive results was observed in the HCC population recruited in Heilongjiang (46.0%) or in Zhejiang (44.4%), whereas a higher proportion of QFT-GIT-positive results was observed in the DC in Zhejiang (63.4%) compared to those observed among the entire group of HCC (45.8%). Here again, the difference was not significant (*p* = 0.10), which might be linked to the relatively small number of control individuals recruited in both provinces. Thus, the difference of sensitivity of both the TST and QFT-GIT between the individuals in the control group was not associated with the province of enrolment, but it was related to the origin of the control individuals. The difference of sensitivity of the two tests is linked to the higher number of DC with LTBI compared to the HCC with LTBI included.

### IGRA in high TB burden countries

As shown in the systematic review and meta-analysis by Sester *et al*. *[* 6] the pooled sensitivity (80.0%, 95%CI: 75–84) for patients with ATB (culture confirmed or non-confirmed by culture) was identical with our results (overall sensitivity: 80.9%). In contrast, our overall specificity (47%, 95%CI: 37–57) was again significantly lower than the pooled specificity (79%; 95%CI: 75–82) described in the meta-analysis. However, the specificity was highly variable depending on the study reported, ranging from 54% to 100% [6]. Recently, in Cape Town (South Africa), Ling *et al*. [25] assessed the accuracy of two IGRAs (QFT-GIT and T-SPOT.TB) among 395 patients (27% were HIV-infected) with suspected TB. The IFN-γ responses were significantly higher in the ATB than in the non-ATB groups (p<0.0001), and the QFT-GIT sensitivity was 76% (95% CI: 68–83%) with a specificity for active TB of 42% (95% CI: 36–49%), respectively [25]. These results are comparable to ours.

To note: in the studies reporting a low level of specificity, the QFT-GIT has been assessed in patients suspected of PTB in whom ATB has been excluded (SM-negative, clinical and radiological findings with a well-defined diagnosis) and considered as true negative controls of ATB disease. Thus, the low specificity of QFT-GIT for ATB diagnosis is linked to the high proportion of positive IGRA results compatible with a diagnosis of LTBI. Unlike low-risk controls, this population of non-TB patients is more representative of patients who would be tested in routine clinical settings.

### Impact of severe TB disease on QFT-GIT sensitivity

The inconsistent values of sensitivity reported in the systematic reviews may be a consequence of a significant heterogeneity between the patients included and study results: advanced TB, co-morbidity, high versus low burden settings [6]. The poor IFN-γ response and a high bacillary load in the PTB patients may be partly related to a T-cell compartmentalization and immunosuppressive mediators including IL-10, TGF-β and regulatory T-cells [26]. Possibly, the memory T-cells that develop into effector-memory T-cells could be concentrated in the lungs [27], being removed from the blood sampled compartment, and those with the most extensive disease may have the greatest attenuation of RD-1-specific Th1 immunity [28]. However, our QFT-GIT results showed that there was no decrease of the median IFN-γ levels (Fig 5) and sensitivity (Table 9) in the group of PTB patients with the highest bacillary load, such as those with a positive SM grade (P4+) compared to those with lower grades. Similar results have been recently reported in South Africa by Theron *et al*. [29] and in India by Lagrange *et al*. [30], where the T-cell IFN-γ responses did not correlate with several measures of the bacterial yield in the sputum of the PTB patients at diagnosis, including SM status and grade, and time to positivity of the liquid culture.

### Impact of HIV infection on QFT-GIT sensitivity

Because only one of our PTB patients was HIV-infected, it is unlikely that the HIV-associated anergy led to a decreased sensitivity in our study. Interestingly, the HIV-TB co-infected patient had an indeterminate QFT-GIT result and there was no significant difference when the sensitivity included or excluded the indeterminate QFT-GIT. The other PTB patient with an indeterminate QFT-GIT result has not been evaluated for his HIV status.

The overall prevalence of HIV remains low in China (0.058% at the end of 2011); however, geographic distribution of the overall 780,000 people living with HIV (including 154,000 AIDS cases) has revealed regions of especially high prevalence [31]. The prevalence of HIV infection in the 2 provinces of recruitment seems to be very low and therefore it is unlikely that it influenced the diagnostic value of the test.

#### TST

In the present study in Zhejiang, a normally distributed TST response (or bimodal distribution) was observed in both the PTB and control group, showing a very small number of measurable reactions between 0 and 10 mm. This suggests that populations demonstrating these results have a very low number of false-positive results, and that the TST is highly specific and might indicate a very low prevalence of NTM infections in this setting (Fig 1B). The second peak observed among the control individuals (10–15 mm) might represent a persisting TST-positive response, being either secondary to a BCG revaccination or associated with a recent LTBI. These conditions should be differentiated using the QFT-GIT [6]. In contrast, in Heilongjiang, a unimodal distributed TST response was observed among the controls showing a single peak at 5 mm, and no clear separation of positive and negative test was observed among the PTB patients. Several cut-off points were needed to assess the diagnostic values of the TST. Similar results to those observed in Heilongjiang have been described earlier by Bass [32] and suggested a high frequency of NTM sensitization in this population.

In Zhejiang, the bimodal distribution of the TST results in the controls showed an antimode at 5 mm that reinforces the validity of the chosen TST cut-off point in the PRC for diagnosing LTBI [15]. This cut-off point revealed a sensitivity for PTB of 98.7%, and a specificity of 26.8%, calculated from the results obtained in DC individuals (Table 4). The cut-off point at 10 mm did not significantly decrease the TST sensitivity for PTB (97.3%; *p* = 0.68); although it increased the specificity (36.6%; *p* = 0.47), but not significantly. Choosing a cut-off point at 15 mm, significantly decreased the TST sensitivity (89.3%; p = 0.0009) and significantly increased the specificity (61.0%; p = 0.0035). In Heilongjiang, a significant decrease of the TST sensitivity for PTB was noticed (Table 5), when the cut-off point increased from 5 mm to 10 mm (72.2% to 59.4%; *p* = 0.038) and from 5 mm to 15 mm (72.2% to 25.6%; *p*<0.0001). Such changes were consequently associated with a significant increase of the specificity for PTB, when calculated from the results obtained in the whole group of HCC: with a 10 mm cut-off point the specificity increased from 40.7% to 62.7% (p = 0.0266) and from 40.7% to 83.1% (p<0.0001) with a 15 mm cut-off point.

Our results show a higher global proportion of LTBI among the included controls (as estimated of the TST positive rate using a 5 mm cut-off point) compared to results reported in several studies performed in China [10,14]. On one hand, this difference could be related to the varying proportions of LTBI among the control populations recruited at different settings, as shown above with the QFT-GIT results. However, the impact of the region of recruitment on the TST results was negligible in our study: the HCC from the 2 provinces presented comparable TST-positive results (60.0% and 55.6%). In contrast, a higher TST-positive rate was observed in the DC individuals (73.2%) than in the HCC (55.6%) recruited in the same province (Zhejiang), or in the entire group of HCC recruited in both provinces (59.3%), but the difference was not significant, which might be linked to the relatively small number of control individuals recruited in both provinces. On the other hand, the higher TST-positive results among our HCC compared to those shown in Feng‘s study might either be related to a different cut-off point (5 mm), or the strength of the tuberculin used [11]. In his study, Feng used a Tuberculin at 5 TU with a 10 mm cut-off point and the TST-positive rate was 38.5%, equivalent with our HCC population using a 10 mm cut-off point (37.3%). Very similar results were observed in Zhang’s study, where the TST-positive rate was 34% in his healthy control population when using a 10 mm cut-off point; this proportion increased to 62.3% with a 5 mm cut-off point [10].

Evaluating the results worldwide, in the last systematic and meta-analysis review assessing the diagnostic values of the TST for PTB, the pooled estimate of sensitivity was 77%, ranging from 66% to 100%, and its specificity in non-BCG-vaccinated populations was consistently high, with a pooled estimate of 97% [33]. However, its pooled estimate was lower in the BCG-vaccinated populations and highly heterogeneous, ranging from 35% to 79% [33].

In our study, the overall TST sensitivity for diagnosing PTB was 86.2% with a specificity of 26.8% as calculated using the DC individuals, but it increased to 40.7% when calculated using the HCC population. The frequency of LTBI in the HCC (community-based) population using a 5 mm cut-off point was 59.3% and decreased as the chosen cut-off point increased. With a cut-off point of 10 mm, as recommended by the other agencies, the LTBI prevalence dropped to 37.3%, and to 16.9% with a 15 mm cut-off point. The very low overall TST specificity observed in our study might be associated with the extremely high coverage (99%) of BCG vaccination in the population of the PRC [20] and/or with Non-Tuberculosis Mycobacteria (NTM) sensitization [19]. However, NTM is not a clinically important cause of false-positive TST, except in populations with a high prevalence of NTM sensitization and a very low prevalence of TB infection [18]. This might be not the case in our tested population in the PRC, as shown by the similar prevalence of LTBI detected by the QFT-GIT or TST and the normally distributed TST responses in PTB and control individuals in Zhejiang.

Thus, the very high coverage of BCG vaccination among the tested population remains the likely explanation, with the exception of those who had received only one BCG vaccination at birth (Table 10), as indicated by Pai *et al*. [34] showing that the BCG given at birth did not influence the TST or IGRA results. Our results confirm the relative weight of the BCG vaccination schedule upon the discordant results. It is worthy to note the complete absence of discordant TST+/QFT-results in individuals who received one BCG at birth (Table 10), and only one individual in the third group (No BCG vaccination). In contrast, in the second group, 33 individuals presented discordant results with a very high proportion of TST+/QFT-GIT- results (69.7%). This might confirm the persisting impact of two BCG vaccinations on the TST, when given at birth and one year later.

#### QFT-GIT versus TST

Although the IGRA and TST are believed to measure the cellular immune responses to *M*. *tuberculosis* antigens, they differ by several means [35]. The TST measures *in vivo* a delayed skin inflammatory response to multiple antigens involving a coordinated response with several cellular compounds and mediators. The IGRA measures *ex vivo* the IFN-γ production by the circulating effector-memory lymphocytes in response to stimulation with a few specific antigens. Because the 2 tests measure related but different biological phenomena, discordant results are often recorded in studies comparing the IGRA and TST [34, 36].

The predictive values of the QFT-GIT for diagnosing PTB were almost identical to those reported for the TST, whatever TST cut-off was used (Table 4). This is linked to a slightly lower sensitivity of the QFT-GIT compared to the TST, but a slightly higher specificity of the QFT-GIT compared to the TST. However, in our study, the very low specificity of the QFT-GIT calculated using the DC impacted negatively the possible usefulness of this assay for ruling PTB in or out; this is linked to the absence of discrimination between PTB and LTBI [21]. Considering a control population with a lower LTBI prevalence (such as the HCC), for assessing the overall diagnostic values of the QFT-GIT and TST for diagnosing PTB, we found a greater NPV and a higher LR for a negative test (Table 5), but our results were still lower than those obtained using either the QFT-GIT [13] or the TSPOT [10, 11] in the PRC. However when we compared the diagnostic values of the QFT-GIT and TST for PTB according to the province of recruitment, significantly better results were observed among the populations in Zhejiang than in those in Heilongjiang.

A high overall agreement between the QFT-GIT and TST was noticed in PTB patients (74.4%), but varied according with the province of recruitment; a significantly lower agreement was noticed in Heilongjiang (62.4%) than in Zhejiang (86.6%), when the TST cut-off point used was 5 mm (p<0.0001). The overall agreement was also low in the entire group of controls (65.0%), with no significant impact according to their origin: the agreement did not differ significantly between the HCC and DC (*p* = 0.83). To note: the calculated agreement tended to decrease both in the PTB and in the controls when higher cut-off points for the TST were used to evaluate positive results; the best agreement was obtained with the 5 mm cut-off point. This might be related to the relative independence between the *in vivo* and *in vitro* assays.

Our study showed a comparable proportion of discordant results (29%), as those described in the systematic review by Menzies *et al*. [33]. Similarly, we observed that around two-thirds of the individuals with discordant results were TST+/IGRA- prevailing both in the PTB patients (66%) and control individuals (60%), as already described [17]. As mentioned, the impact of the BCG vaccination enlightens mostly the false-positive TST [37], because the QFT-GIT uses only *M*. *tuberculosis-*specific antigens, whereas the tuberculin is a mix of about 200 antigens from *M*. *tuberculosis* that are shared with all NTM, as well as with all the strains developed from *M*.*bovis* used for the Bacille Calmette-Guérin (BCG) vaccination [34,35]. Additionally, a higher TST-positive rate compared to the QFT-GIT-positive rate might also indicate that the TST is more likely to detect a resolved or old LTBI while the QFT-GIT mainly detects current or recent infections [38,39]. Another possible explanation of the high TST-positive rate in our study was the use of the recommended low cut-off point (at 5 mm) for diagnosing LTBI. In fact, using a TST cut-off point at 15 mm significantly decreased the proportion of the TST+/QFT-GIT- discordant results, both among the PTB patients (p<0.0001) and controls (p<0.0001). This confirms the hypothesis that the prevalence of these discordant results should decrease with a higher cut-off point [11] and that new recommendations for the TST in diagnosing PTB should be discussed.

Little attention has been given in the literature to the TST-negative but QFT-GIT-positive discordant results, but assuming that the QFT-GIT is highly specific, it is likely that this combination indicates a recent LTBI [40]. Another factor has been suggested: waning of the TST with age in PTB patients [41]. Indeed, our results (see S1 Fig) showed such a significant waning of the TST with age in the PTB patients (p = 0.009), but the inverse was observed in the controls where a significant increase of TST with age was observed (p = 0.04). Whether the QFT-GIT wanes with age to the same extent as the TST is an open question, and varying results have been reported [42, 43]. As shown in our study, waning of the individual INF-γ production was associated with age, both in the PTB patients and controls (see S2 Fig), but was only significant in the PTB (p = 0.0043). Similar results have been described in a Japanese study showing a close association between age and LTBI for the QFT-GIT and to a lesser degree for the TST [41]. To date, the immunological interpretation of this observation is not clear. Furthermore, our study also showed that the median age was significantly higher in the QFT-GIT-negative (p = 0.04) and TST-negative (p = 0.06) PTB patients compared to those with positive tests (see S1 Fig). The waning of the specific interferon-γ response after years of TB infection was also described in a Japanese population based on estimates of the expected prevalence of LTBI [42]. In addition, Arend [43] reported that the TST-/QFT-GIT+ discordant results are associated with several indicators of a recent *M*. *tuberculosis* exposure. Likewise, two other studies showed that a high proportion of persons with the discordant TST-/QFT-GIT+ reactions have had a spontaneous QFT-GIT reversion [44, 45]. This suggests that an exposure to *M*. *tuberculosis* could lead to infection, which might be then cleared as suggested by Anderson [35]. It also suggests that the QFT-GIT may provide a more quantitative and dynamic measurement of cellular immune response than the TST, and would be important for serial testing studies after defining the appropriate cut-off point. Our study showed that the waning was higher for the QFT-GIT than for the TST. The hypothesis that the TST could be more reactive to old infections while the QFT-GIT, mainly indicating recent infections, should assessed in serial testing studies.

#### Cumulative results of smear microscopy, the QFT-GIT and TST

A recent study suggests that the combination of different immunodiagnostic tests may improve their diagnostic accuracy [46]. These authors showed that the 99% combined sensitivity of the TST plus IGRA (ELISPOT) reflects the fact that patients who had a false-negative result with one test were distinct from those who had a false-negative result with the other. This implies that distinct immunological processes underlie failure of these different, yet complementary, immune-based tests and this approach can be used to exclude PTB in patients with a moderate to high pre-test probability of disease.

Two previous IGRA studies in high-burden settings have been conducted among confirmed TB patients. One study from South Africa [47] and another from India [48] reported a QFT-GIT sensitivity of 76% and 91%, respectively, results comparable to those reported here (sensitivity of 80.9% in PTB). Both studies reported that the QFT-GIT plus TST combination achieved a sensitivity of at least 96% and could be useful for excluding active TB. Our study also showed a similar increase of the cumulative sensitivity (96.4%), but was associated with a dramatic decrease of the cumulative specificity (23.0%) with a suboptimal LR for positive test (1.25) but with a significant increase of the LR for the negative test (6.39), which may be the best available option to rapidly exclude PTB by immunodiagnostic tests.

A previous study carried out in India has shown that the cumulative results of the smear microscopy and the 2 immunological tests improved the detection rate of ATB [30]. Similarly, our study shows a cumulative sensitivity of 97.5% with an increase of the LR for the negative test (9.2), indicating that such triple association may rule out PTB in those who are negative for all 3 tests.

In this study, the diagnosis of PTB was performed using the regular recommendations of the Center for Disease Control and Prevention’s China TB control program [2] that include chest X-rays and SM, available at the county laboratory level. However, no culture of the sputum specimens was done for confirmation [2], which is common in China since there are few reference laboratories where the *M*. *tuberculosis* culture can be performed. Knowing this pitfall for proper TB diagnosis, a parallel study was undertaken in the same counties of the 2 provinces, looking at the respective diagnostic values of the classical ZN test compared to the solid culture using the Lowenstein Jensen (LJ) medium. Preliminary results (Lagrange PH, manuscript in preparation) indicate that among the 160 PTB patients included, the cumulative SM result of the 3 specimens was positive in 84/160, yielding an overall SM sensitivity of 52.5% (95% CI: 44.5–60.4), which was not significantly higher compared to the SM in the present study (*p* = 0.062) with a specificity of 100% (95% CI: 94.5–100). The solid culture was positive in 86/160 that yielded an overall culture sensitivity of 53.8% (95% CI: 45.7–61.7). The agreement between the SM and the culture was 87.5% (95% CI: 81.4–92.2), with a good k coefficient (0.749). The contamination rate of the culture was very low (8 on 480 slants: 1.7%). However, even if microbiological confirmation by either culture and/or highly sensitive molecular tests remains the gold standard, in our 2 studies performed in the same counties of the 2 provinces, the SM test performed equally well as the culture. Very similar results were obtained in a recently published study done in China comparing the diagnostic accuracy of SM to that of the solid culture using the LJ medium [49]

## Conclusions

Our study confirms that neither the QFT-GIT,or TST alone can be used to rule in active TB in areas with a high prevalence of LTBI. These assays should also not be used to rule out TB disease when performed alone due to their suboptimal sensitivity and low NPV [50]. Thus, the present commercially available version of the QFT-GIT has a very limited role, if any, in the diagnosis of active PTB. However, when combined with SM, the QFT-GIT may be a reliable and useful tool to rule out active TB. Although a similar result could be achieved with a chest X-ray, these findings have great relevance for clinical practice in high-burden, low-resource settings and are consistent with recent WHO recommendations on IGRAs in low- and middle-income countries [51].

## Supporting Information

S1 Fig Age distribution among the enrolled patients and controls stratified by QFT and TST. Individual age among all the PTB patients stratified by (A) QFT-GIT and TST (B) status, and in the control individuals stratified by (C) QFT-GIT and TST (D) status. Continuous lines represent the median and interquartile range (25%-75%). **Abbreviations:** QFT: QuantiFERON TB Gold In-Tube; TST: tuberculin skin test; PTB: pulmonary tuberculosis.
(TIF)

S2 Fig Impact of age on the individual QFT-GIT responses in the enrolled patients and controls. Impact of age (in years) on the individual QFT-GIT responses (A) in the entire group of controls (R^2^ = 0.006077; p = 0.44), (C) in all active PTB patients (R^2^ = 0.02704; p = 0.0043), or on the TST individual responses in (B) the entire group of controls (R^2^ = 0.4071; p = 0.0441), and (D) all active PTB patients (R^2^ = 0.02373; p = 0.0095). Continuous lines represent the median and hatched lines represent the interquartile range (25%-75%). **Abbreviations:** PTB: pulmonary tuberculosis; QFT-GIT: QuantiFERON TB Gold In-Tube; TST: tuberculin skin test.
(TIF)

S1 File Impact of age on the QFT-GIT and TST status. Relationship between age and the QFT-GIT and TST individual results. Table A in S1 File. Analysis of the combination of tests evaluated among 300 active PTB patients and 100 Controls concomitantly tested and stratified by province of enrolment (with a TST using a 5 mm cut-off point). Table B in S1 File. p values of multiple comparisons among tests evaluating sensitivity for active TB in active PTB patients (as reported in Table A). All TB patients (both provinces). Table C in S1 File. Analysis of the combination of tests evaluated among 300 active PTB patients and 100 Controls concomitantly tested and stratified by province of enrolment (with a TST using a 10 mm cut-off point). Table D in S1 File. p values of multiple comparisons among tests evaluating sensitivity for active TB in active PTB patients (as reported in Table C). All TB patients (both provinces). Table E in S1 File. Analysis of the combination of tests evaluated among 300 active PTB patients and 100 Controls concomitantly tested and stratified by province of enrolment (with a TST using a 15 mm cut-off point). Table F in S1 File. p values of multiple comparisons among tests evaluating sensitivity for active TB in active PTB patients (as reported in Table E in S1 File). All TB patients (both provinces).
(DOC)
