# Supplementary figures and images for: Diagnostic Values of the QuantiFERON-TB Gold In-Tube Assay Carried out in China for Diagnosing Pulmonary Tuberculosis
Source: PLoS One. 2015 Apr 13;10(4):e0121021. doi: 10.1371/journal.pone.0121021 (PMC4395092; doi:10.1371/journal.pone.0121021)

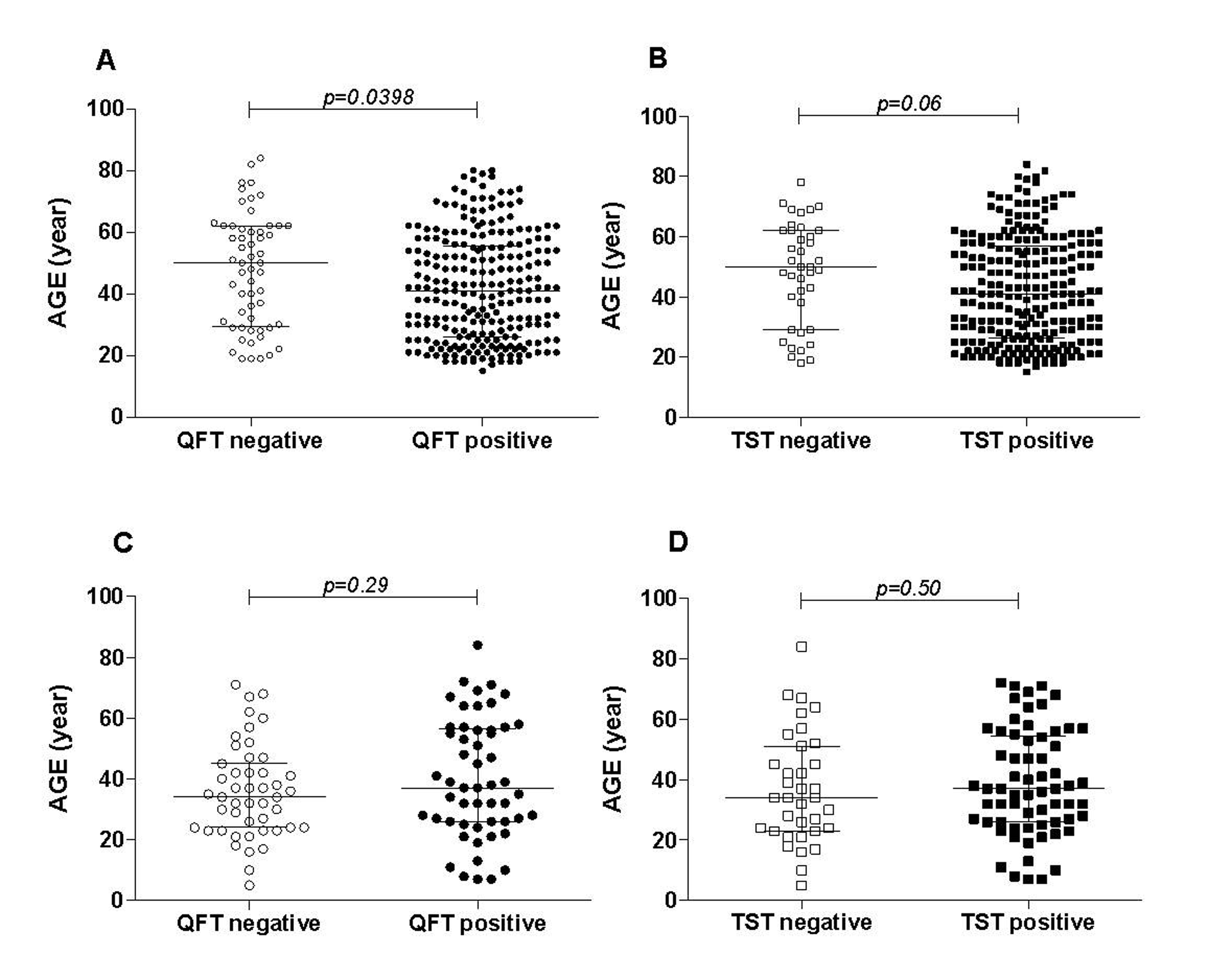

Supplement: S1 Fig — Individual age among all the PTB patients stratified by (A) QFT-GIT and TST (B) status, and in the control individuals stratified by (C) QFT-GIT and TST (D) status. Continuous lines represent the median and interquartile range (25%-75%). Abbreviations: QFT: QuantiFERON TB Gold In-Tube; TST: tuberculin skin test; PTB: pulmonary tuberculosis. (TIF) [file pone.0121021.s001.tif]

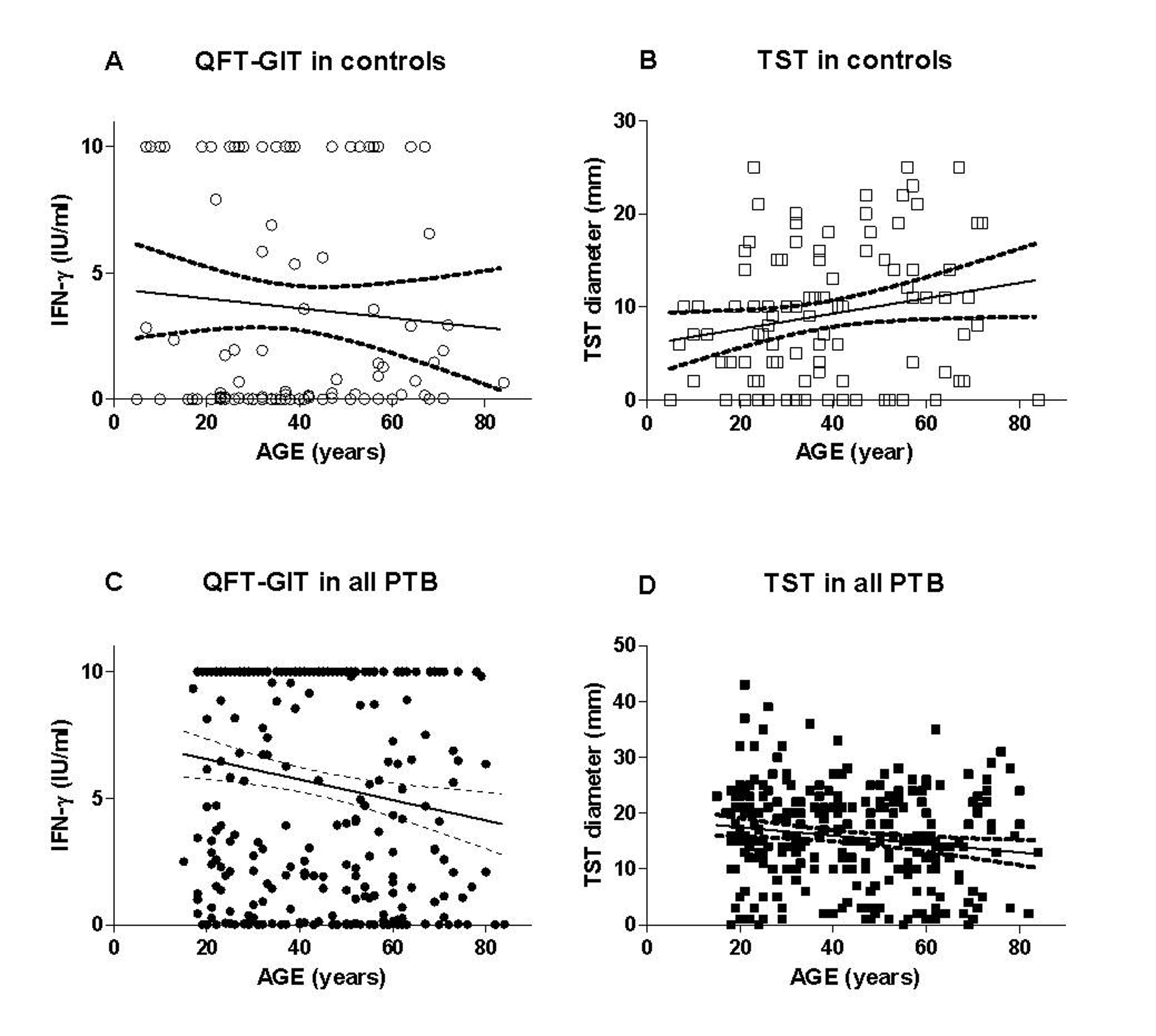

Supplement: S2 Fig — Impact of age (in years) on the individual QFT-GIT responses (A) in the entire group of controls (R2 = 0.006077; p = 0.44), (C) in all active PTB patients (R2 = 0.02704; p = 0.0043), or on the TST individual responses in (B) the entire group of controls (R2 = 0.4071; p = 0.0441), and (D) all active PTB patients (R2 = 0.02373; p = 0.0095). Continuous lines represent the median and hatched lines represent the interquartile range (25%-75%). Abbreviations: PTB: pulmonary tuberculosis; QFT-GIT: QuantiFERON TB Gold In-Tube; TST: tuberculin skin test. (TIF) [file pone.0121021.s002.tif]
