# Supplementary material for: Diagnostic Values of the QuantiFERON-TB Gold In-Tube Assay Carried out in China for Diagnosing Pulmonary Tuberculosis
Source: PLoS One. 2015 Apr 13;10(4):e0121021. doi: 10.1371/journal.pone.0121021 (PMC4395092; doi:10.1371/journal.pone.0121021)
Supplement: S1 File — Relationship between age and the QFT-GIT and TST individual results. Table A in S1 File. Analysis of the combination of tests evaluated among 300 active PTB patients and 100 Controls concomitantly tested and stratified by province of enrolment (with a TST using a 5 mm cut-off point). Table B in S1 File. p values of multiple comparisons among tests evaluating sensitivity for active TB in active PTB patients (as reported in Table A). All TB patients (both provinces). Table C in S1 File. Analysis of the combination of tests evaluated among 300 active PTB patients and 100 Controls concomitantly tested and stratified by province of enrolment (with a TST using a 10 mm cut-off point). Table D in S1 File. p values of multiple comparisons among tests evaluating sensitivity for active TB in active PTB patients (as reported in Table C). All TB patients (both provinces). Table E in S1 File. Analysis of the combination of tests evaluated among 300 active PTB patients and 100 Controls concomitantly tested and stratified by province of enrolment (with a TST using a 15 mm cut-off point). Table F in S1 File. p values of multiple comparisons among tests evaluating sensitivity for active TB in active PTB patients (as reported in Table E in S1 File). All TB patients (both provinces). (DOC) [file pone.0121021.s003.doc]

**Supporting Information**

**Impact of age on the QFT-GIT and TST status**

The median (IQR) age was calculated in the entire PTB group and control group stratified by QFT-GIT and TST status (using a 5mm cut-off point).The results are shown in **S1 Fig.** Among the PTB, the median (IQR) age was significantly higher in QFT-GIT-negative patients (50.0 years: 29.5-62.0) compared to QFT-GIT-positive (41.0 years: 25.5-54.5) patients (p=0.0398) (**S1A Fig.**). Similarly, the median (IQR) age was higher in the TST-negative (50.0 years: 29.0-62.0) than in the TST-positive PTB patients (41.0 years: 26.25-56.75), but the difference was not significant (p=0.0675) (**S1B Fig.**).

Among the control individuals, the median (IQR) age was lower in QFT-GIT-negative (34.0 years: 24-45) than in the QFT-GIT-positive (37 years: 26-56.5) individuals, but the difference was not significant (p=0.29) (**S1C Fig.**). Similarly, the median (IQR) age was lower in the TST-negative (34 years: 23-51) than in the TST-positive (37 years: 26-54.5) individuals, but the difference was not significant (p=0.50) (**S1D Fig.**).

Among all included individuals, the median age (IQR) was identical in the QFT-GIT-positive (40.0 years: 28-58) and QFT-GIT-negative (40.0 years: 26-56) (p=0.77). The median age (IQR) was higher in the TST-negative (43 years: 25.5-58.5) compared to TST-positive (40.0 years: 26-56), but the difference was not significant (p=0.57) (data not shown).

**Relationship between age and the QFT-GIT and TST individual results**

The correlation between the individual QFT-GIT results (IU/ml) or TST diameter (in mm) with age was further analyzed among all the PTB and all the controls; the results are shown in **S2 Fig**. The individual QFT-GIT level was not associated with the age of the controls (R^2^= 0.006077, p=0.44), showing a slight tendency of the INF-γ level (slope: -0.019±0.024) to decrease with age (**S2A Fig.**). Yet in the PTB patients, a significant inverse correlation was observed with the individual QFT-GIT level and age (R^2^= 0.02704; p=0.0043), showing a decrease of the INF-γ level (slope: -0.040±0.014) with increasing age (**S2C Fig.**). In contrast, the individual TST diameter was significantly correlated with the age in the controls (R^2^= 0.04071, p=0.044), showing a significant increase of the TST diameter (slope: +0.0826±0.0405) with age (**S2B Fig.**). In the PTB, the individual TST diameter was also significantly associated with age (R^2^= 0.02373, p=0.009), but an inverse correlation was noticed, showing a decrease of the TST diameter (slope:-0.07492±0.0286) with age (**S2D Fig.**).

We assessed whether the combination of tests improves their respective sensitivity and specificity for ATB diagnosis among the 281 PTB patients and the 100 controls who were concomitantly tested with the smear microscopy, QFT-GIT and TST, using 3 different cut-off points (**Tables A-F**).

Using a TST with a 5 mm cut-off point, the overall sensitivity was significantly different among the different tests when assessed as single tests and combined tests (p<0.0001).It is worthy to note that the sensitivity of the QFT-GIT or TST was significantly higher than the SM sensitivity (p<0.0001), independent of the province of enrolment (**Table A**). Moreover, the cumulative sensitivity of the QFT-GIT plus TST, associated or not with the SM, was significantly higher than any of the single tests when evaluated in all PTB (p<0.0001) and in those in Heilongjiang (p<0.0001) (**Table B**). It is worthy to note that the cumulative sensitivity of the QFT-GIT plus TST, associated or not with the SM, was 100.0% among the PTB in Zhejiang, with a significant difference for the SM alone (p<0.0001) or for the QFT-GIT alone (p<0.0001), but the difference with the TST alone (98.7%) was not significant (p=0.49). These results indicate that in PTB, the combination of the available SM with immunological tests did increase the possibility of detecting PTB and was possibly important to rule out active TB in suspected patients with all negative tests.

On the other hand, this increase of cumulative sensitivity was associated with a concomitant decrease of the cumulative specificity (**Table B**). The cumulative results of the QFT-GIT plus the TS, with a 5 mm cut-off point, were positive in 77 controls that yielded an overall significantly lower specificity (23.0%) compared to those of the QFT-GIT (p=0.0006) alone, but it was not statistically different compared to the TST (p=0.0860) alone. Using a 10 mm or 15 mm cut-off point, the TST specificity increased with a concomitant decrease of sensitivity (**Tables C-D**). Nevertheless, the overall sensitivity was still significantly different among the different tests when evaluated as single tests and combined tests (p<0.0001), and the cumulative sensitivity of the QFT-GIT assay plus the TST, associated or not with SM, was significantly higher than those of the single test when evaluated in the whole group of PTB (**Tables E-F**).

**Table A**: Analysis of the combination of tests evaluated among 300 active PTB patients and 100 Controls concomitantly tested and stratified by province of enrolment (with a TST using a 5 mm cut-off point).

|  | **Positive over total tested** | | | | | | | | **Negative over total tested** | | | | | |
| --- | --- | --- | --- | --- | --- | --- | --- | --- | --- | --- | --- | --- | --- | --- |
|  | **Percentage (95% CI)** | | | | | | | | **Percentage (95% CI)** | | | | | |
|  | **Sensitivity** | | | | | | | | **Specificity** | | | | | |
|  | **SM** | **TST** | **QFT** | **QFT with TST** | **SM with TST** | **SM with QFT** | **QFT with TST with SM** | *p value** | **SM** | **TST** | **QFT** | **QFT with TST** | **QFT with TST with SM** | *p value****** |
| **All PTB patients** | 129/300 | 244/283 | 241/298 | 271/281 | 269/283 | 263/298 | 274/281 |  | 100/100 | 35/100 | 47/100 | 23/100 | 23/100 |  |
|  | **43.0** | **86.2** | **80.9** | **96.4** | **95.1** | **88.3** | **97.5** | *<0.0001* | **100.0** | **35.0** | **47.0** | **23.0** | **23.0** | *<0.0001* |
|  | (37.3-48.8) | (81.7-90.0) | (75.9-85.2) | (93.6-98.3) | (91.8-97.3) | (84.1-91.7) | (94.9-99.0) |  | (96.4-100) | (25.7-45.2) | (36.9-57.2) | (15.2-32.5) | (15.2-32.5) |  |
|  |  |  |  |  |  |  |  |  |  |  |  |  |  |  |
| **Heilongjiang** | 79/150 | 96/133** | 113/150 | 123/133 | 121/133 | 126/150 | 126/133 |  | 50/50 | 20/50 | 27/50 | 15/50 | 15/50 |  |
|  | **52.7** | **72.2** | **75.3** | **92.5** | **91.0** | **84.0** | **94.7** | *<0.0001* | **100.0** | **40.0** | **54.0** | **30.0** | **30.0** | *<0.0001* |
|  | (44.4-60.9) | (63.8-79.6) | (67.6-  82.0) | (86.6-96.3) | (84.8-95.3) | (77.1-89.5) | (89.5-97.9) |  | (92.9-100) | (26.4-54.8 | (39.3-68.2) | (17.9-44.6) | (17.9-44.6) |  |
|  |  |  |  |  |  |  |  |  |  |  |  |  |  |  |
| **Zhejiang** | 50/150 | 148/150 | 128/148*** | 148/148 | 148/150 | 137/148 | 148/148 |  | 50/50 | 15/50 | 20/50 | 8/50 | 8/50 |  |
|  | **33.3** | **98.7** | **86.5** | **100.0** | **98.7** | **92.6** | **100.0** | *<0.0001* | **100.0** | **30.0** | **40.0** | **16.0** | **16.0** | *<0.0001* |
|  | (25.9-41.5) | (95.3-99.8) | (79.9-  91.6) | (97.5-100) | (95.3-99.8) | (87.1-96.2) | (97.5-100) |  | (92.9-100) | (17.9-44.6) | (26.4-54.8) | (7.2-29.1) | (7.2-29.1) |  |

**Footnotes:** PTB: pulmonary tuberculosis; TST: tuberculin skin test (with a 5 mm cut-off point); QFT: QuantiFERON® Gold In-Tube (indeterminate results are included as negative results); SM: smear microscopy.

* Χ square test was used for statistical analysis. ** 17 patients did not return for TST readings. *** 2 patients had indeterminate QFT-GIT results.

**Table B**: **p values of multiple comparisons among tests evaluating sensitivity for active TB in active PTB patients (as reported in S1 Table)**

All TB patients (both provinces)

|  | **Single test** | | | **Combined tests** | | | |
| --- | --- | --- | --- | --- | --- | --- | --- |
|  | **TST** | **QFT-GIT** | **SM** | **TST+QFT-GIT** | **TST+SM** | **QFT-GIT+SM** | **QFT-GIT+SM+TST** |
| **TST** | NA | 0.0938 | <0.0001 | <0.0001* | 0.0004* | 0.5339 | **<0.0001**** |
| **QFT-GIT** | - | NA | <0.0001 | <0.0001* | <0.0001* | 0.0169 | **<0.0001**** |
| **SM** | - | - | NA | <0.0001* | <0.0001* | **<0.0001*** | **<0.0001**** |
| **TST+QFT-GIT** | - | - | - | NA | 0.5324 | **0.0003*** | 0.6237 |
| **TST+SM** | - | - | - | - | NA | 0.0042 | 0.1810 |
| **QFT-GIT+SM** | - | - | - | - | - | NA | **<0.0001**** |
| **Heilongjiang** |  | | |  | | | |
|  | **Single test** | | | **Combined tests** | | | |
|  | **TST** | **QFT-GIT** | **SM** | **TST+QFT-GIT** | **TST+SM** | **QFT-GIT+SM** | **QFT-GIT+SM+TST** |
| **TST** | NA | 0.5889 | 0.0009 | <0.0001* | 0.0001* | 0.0202 | **<0.0001**** |
| **QFT-GIT** | - | NA | <0.0001 | <0.0001* | 0.0005* | 0.0846 | **<0.0001**** |
| **SM** | - | - | NA | <0.0001* | <0.0001* | **<0.0001*** | **<0.0001**** |
| **TST+QFT-GIT** | - | - | - | NA | 0.8244 | 0.0427 | 0.6174 |
| **TST+SM** | - | - | - | - | NA | 0.1069 | 0.3413 |
| **QFT-GIT+SM** | - | - | - | - | - | NA | **0.0040**** |
| **Zhejiang** |  | | |  | | | |
|  | **Single test** | | | **Combined tests** | | | |
|  | **TST** | **QFT-GIT** | **SM** | **TST+QFT-GIT** | **TST+SM** | **QFT-GIT+SM** | **QFT-GIT+SM+TST** |
| **TST** | NA | <0.0001 | <0.0001 | 0.4983 | 1.000 | 0.0108 | 0.4983 |
| **QFT-GIT** | - | NA | <0.0001 | <0.0001* | <0.0001* | 0.1278 | **<0.0001**** |
| **SM** | - | - | NA | <0.0001* | <0.0001* | **<0.0001*** | **<0.0001**** |
| **TST+QFT-GIT** | - | - | - | NA | 0.4983 | **0.0008*** | 1.000 |
| **TST+SM** | - | - | - | - | NA | 0.0108 | 0.4983 |
| **QFT-GIT+SM** | - | - | - | - | - | NA | **0.0008**** |

**Footnote:** PTB: tuberculosis; TST: tuberculin skin test (with a 5 mm cut-off point); QFT-GIT: QuantiFERON® Gold In-Tube (indeterminate results are included as negative results; SM: smear microscopy.

* Significant after Bonferroni correction for 5 multiple comparisons, p value≤0.01; ** significant after Bonferroni correction for 7 multiple comparisons, p value≤0.007

**Table C**: **Analysis of the combination of tests evaluated among 300 active PTB patients and 100 Controls concomitantly tested and stratified by province of enrolment (with a TST using a 10 mm cut-off point)**

|  | **Positive over total tested** | | | | | | | | **Negative over total tested** | | | | | |
| --- | --- | --- | --- | --- | --- | --- | --- | --- | --- | --- | --- | --- | --- | --- |
|  | **Percentage (95% CI)** | | | | | | | | **Percentage (95% CI)** | | | | | |
|  | **Sensitivity** | | | | | | | | **Specificity** | | | | | |
|  | **SM** | **TST** | **QFT** | **QFT with TST** | **SM with TST** | **SM with QFT** | **QFT with TST with SM** | ***p value**** | **SM** | **TST** | **QFT** | **QFT with TST** | **QFT with TST with SM** | **p value*** |
| **All TB patients** | 129/300 | 225/283 | 241/298 | 265/281 | 246/283 | 263/298 | 269/281 |  | 100/100 | 52/100 | 47/100 | 30/100 | 30/100 |  |
|  | **43.0** | **79.5** | **80.9** | **94.3** | **86.9** | **88.3** | **95.7** | *<0.0001* | **100.0** | **52.0** | **47.0** | **30.0** | **30.0** | *<0.0001* |
|  | (37.3-48.8) | (74.3-84.1) | (75.9-85.2) | (90.9-  96.7) | (82.4-  90.6 | (84.1-91.7) | (92.7-97.8) |  | (96.4-100) | (41.8-62.1 | (36.9-57.2) | (21.2-40.0) | (21.2-40.0) |  |
|  |  |  |  |  |  |  |  |  |  |  |  |  |  |  |
| **Heilongjiang** | 79/150 | 79/133 | 113/150 | 118/133 | 99/133 | 126/150 | 122/133 | *<0.0001* | 50/50 | 33/50 | 27/50 | 21/50 | 21/50 | *<0.0001* |
|  | **52.7** | **59.4** | **75.3** | **88.7** | **74.4** | **84.0** | **91.7** |  | **100.0** | **66.0** | **54.0** | **42.0** | **42.0** |  |
|  | (44.4-60.9) | (50.5-67.8) | (67.6-82.0) | (82.1-  93.6) | (66.2-81.6) | (77.1-89.5) | (85.7-95.8) |  | (92.9-100) | (51.2-78.8) | (39.3-68.2) | (28.2-56.8) | (28.2-56.8) |  |
|  |  |  |  |  |  |  |  |  |  |  |  |  |  |  |
| **Zhejiang** | 50/150 | 146/150 | 128/148*** | 147/148 | 147/150 | 137/148 | 147/148 | *<0.0001* | 50/50 | 19/50 | 20/50 | 9/50 | 9/50 | *<0.0001* |
|  | **33.3** | **97.3** | **86.5** | **99.3** | **98.0** | **92.6** | **99.3** |  | **100.0** | **38.0** | **40.0** | **18.0** | **18.0** |  |
|  | (25.9-41.5) | (93.3-99.3) | (79.9-91.6) | (96.3-  100) | (94.3-99.6) | (87.1-96.2) | (96.3-100) |  | (92.9-100) | (24.6-52.8) | (26.4-54.8) | (8.6-  31.4) | (8.6-  31.4) |  |

**Footnotes: P**TB: tuberculosis; TST: tuberculin skin test (with a 10 mm cut-off point); QFT: QuantiFERON® Gold In-Tube (indeterminate results are included as negative results); SM: smear microscopy.

* Χ square test was used for statistical analysis. ** 17 patients did not return for TST readings. *** 2 patients had indeterminate QFT-GIT results.

**Table D: p values of multiple comparisons among tests evaluating sensitivity for active TB in active PTB patients (as reported in S3 Table)**

All TB patients (both provinces)

|  | **Single test** | | | **Combined tests** | | | |
| --- | --- | --- | --- | --- | --- | --- | --- |
|  | **TST** | **QFT-GIT** | **SM** | **TST+QFT-GIT** | **TST+SM** | **QFT-GIT+SM** | **QFT-GIT+SM+TST** |
| **TST** | NA | 0.7548 | <0.0001 | <0.0001* | 0.0241* | 0.0046 | **<0.0001**** |
| **QFT-GIT** | - | NA | <0.0001 | <0.0001* | 0.0553 | 0.0169 | **<0.0001**** |
| **SM** | - | - | NA | <0.0001* | <0.0001* | **<0.0001*** | **<0.0001**** |
| **TST+QFT-GIT** | - | - | - | NA | 0.0036 | **0.0122** | 0.5616 |
| **TST+SM** | - | - | - | - | NA | 0.7058 | **0.0003**** |
| **QFT-GIT+SM** | - | - | - | - | - | NA | **0.0012**** |
| **Heilongjiang** |  | | |  | | | |
|  | **Single test** | | | **Combined tests** | | | |
|  | **TST** | **QFT-GIT** | **SM** | **TST+QFT-GIT** | **TST+SM** | **QFT-GIT+SM** | **QFT-GIT+SM+TST** |
| **TST** | NA | 0.0050 | 0.2813 | <0.0001* | 0.0131 | <0.0001* | **<0.0001**** |
| **QFT-GIT** | - | NA | <0.0001 | 0.0053* | 0.8913 | 0.0846 | **0.0002**** |
| **SM** | - | - | NA | <0.0001* | 0.0002* | **<0.0001*** | **<0.0001**** |
| **TST+QFT-GIT** | - | - | - | NA | 0.0041* | <0.0001* | 0.5364 |
| **TST+SM** | - | - | - | - | NA | 0.053 | **0.0003**** |
| **QFT-GIT+SM** | - | - | - | - | - | NA | 0.0693 |
| **Zhejiang** |  | | |  | | | |
|  | **Single test** | | | **Combined tests** | | | |
|  | **TST** | **QFT-GIT** | **SM** | **TST+QFT-GIT** | **TST+SM** | **QFT-GIT+SM** | **QFT-GIT+SM+TST** |
| **TST** | NA | 0.0005 | <0.0001 | 0.3708 | 1.000 | 0.0681 | 0.3708 |
| **QFT-GIT** | - | NA | <0.0001 | <0.0001* | <0.0001* | 0.1278 | **<0.0001**** |
| **SM** | - | - | NA | <0.0001* | <0.0001* | **<0.0001*** | **<0.0001**** |
| **TST+QFT-GIT** | - | - | - | NA | 0.6225 | 0.0054 | 1.000 |
| **TST+SM** | - | - | - | - | NA | 0.0302 | 0.6225 |
| **QFT-GIT+SM** | - | - | - | - | - | NA | 0.0054 |

**Footnote:** PTB: pulmonary tuberculosis; TST: tuberculin skin test (with a 10 mm cut-off point); QFT-GIT: QuantiFERON® Gold In-Tube (indeterminate results are included as negative results; SM: smear microscopy.

* Significant after Bonferroni correction for 5 multiple comparisons, p value≤0.01; ** significant after Bonferroni correction for 7 multiple comparisons, p value≤0.007

**Table E: Analysis of the combination of tests evaluated among 300 active PTB patients and 100 Controls concomitantly tested and stratified by province of enrolment** (with a TST using a 15 mm cut-off point)

|  | **Positive over total tested** | | | | | | | | **Negative over total tested** | | | | | |
| --- | --- | --- | --- | --- | --- | --- | --- | --- | --- | --- | --- | --- | --- | --- |
|  | **Percentage (95% CI)** | | | | | | | | **Percentage (95% CI)** | | | | | |
|  | **Sensitivity** | | | | | | | | **Specificity** | | | | | |
|  | **SM** | **TST** | **QFT** | **QFT with TST** | **SM with TST** | **SM with QFT** | **QFT with TST with SM** | *p value** | **SM** | **TST** | **QFT** | **QFT with TST** | **QFT with TST with SM** | *p value****** |
| **All TB patients** | 129/300 | 165/283 | 241/298 | 252/281 | 219/283 | 263/298 | 259/281 |  | 100/100 | 74/100 | 47/100 | 39/100 | 30/100 |  |
|  | **43.0** | **58.3** | **80.9** | **89.7** | **77.4** | **88.3** | **92.2** | *<0.0001* | **100** | **74.0** | **47.0** | **39.0** | **30.0** | *<0.0001* |
|  | (37.3-48.8) | (52.3-  64.1) | (75.9-  85.2) | (85.5-93.0) | (72.1-82.1) | (84.1-91.7) | (88.4-95.0 |  | (96.4-100) | (64.3-82.3) | (36.9-57.2 | (29.4-49.3) | (21.2-  40.0) |  |
| **Heilongjiang** | 79/150 | 34/133** | 113/150 | 107/133 | 80/133 | 126/150 | 114/133 |  | 50/50 | 43/50 | 27/50 | 24/50 | 24/50 |  |
|  | **52.7** | **25.6** | **75.3** | **80.5** | **60.2** | **84.0** | **81.4** | *<0.0001* | **100** | **86.0** | **54.0** | **48.0** | **48.0** | *<0.0001* |
|  | (44.4-60.9) | (18.4-  33.9 | (67.6-  82.0) | (72.7-86.9) | (51.3-68.5) | (77.1-89.5) | (74.0-87.5 |  | (92.9-100) | (73.3-94.2) | (39.3-68.2) | (33.9-62.6) | (33.9-  62.6) |  |
| **Zhejiang** | 50/150 | 131/150 | 128/148*** | 145/148 | 139/150 | 137/148 | 145/148 |  | 50/50 | 31/50 | 20/50 | 15/50 | 15/50 |  |
|  | **33.3** | **87.3** | **86.5** | **98.0** | **92.7** | **92.6** | **98.0** | *<0.0001* | **100** | **62.0** | **40.0** | **30.0** | **30.0** | *<0.0001* |
|  | (25.9-41.5) | (80.9-  92.2) | (79.9-  1.6) | (94.2-99.6) | (87.3-96.3) | (87.1-96.2) | (94.2-99.6) |  | (92.9-100) | (47.2-73.4) | (26.4-54.8) | (17.9-44.6) | (17.9-  44.6) |  |

**Footnotes:** PTB: pulmonary tuberculosis; TST: tuberculin skin test (with a 15 mm cut-off point); QFT: QuantiFERON® Gold In-Tube (indeterminate results are included as negative results); SM: smear microscopy.

* Χ square test was used for statistical analysis. ** 17 patients did not return for TST readings. *** 2 patients had indeterminate QFT-GIT results.

**Table F: *p* values of multiple comparisons among tests evaluating sensitivity for active TB in active PTB patients (as reported in S5 Table)**

All TB patients (both provinces)

|  | **Single test** | | | **Combined tests** | | | |
| --- | --- | --- | --- | --- | --- | --- | --- |
|  |  | | |  | | | |
|  | **TST** | **QFT-GIT** | **SM** | **TST+ QFT-GIT** | **TST+QFT-GIT** | **QFT-GIT+SM** |  |
| **TST** | NA | 0.0001 | 0.0003 | <0.0001* | <0.0001* | <0.0001* | **<0.0001**** |
| **QFT-GIT** | - | NA | <0.0001 | 0.0033* | 0.3087 | 0.0169 | **<0.0001**** |
| **SM** | - | - | NA | <0.0001* | <0.0001* | **<0.0001*** | **<0.0001**** |
| **TST+QFT-GIT** | - | - | - | NA | <0.0001* | 0.5987 | 0.3784 |
| **TST+SM** | - | - | - | - | NA | 0.0006* | **<0.0001**** |
| **QFT-GIT+SM** | - | - | - | - | - | NA | 0.1258 |

|  |  |  |  |  |  |  |  |
| --- | --- | --- | --- | --- | --- | --- | --- |
| **Heilongjiang** | **TST** | **QFT-GIT** | **SM** | **TST+ QFT-GIT** | **TST+QFT-GIT** | **QFT-GIT+SM** |  |
| **TST** | NA | <0.0001 | <0.0001 | <0.0001* | <0.0001* | <0.0001* | **<0.0001**** |
| **QFT-GIT** | - | NA | <0.0001 | 0.3195 | 0.0073* | 0.0846 | 0.3195 |
| **SM** | - | - | NA | <0.0001* | 0.2307 | <0.0001***** | **<0.0001**** |
| **TST+QFT-GIT** | - | - | - | NA | 0.0004* | 0.4404 | 1.000 |
| **TST+SM** | - | - | - | - | NA | <0.0001* | **0.0004**** |
| **QFT-GIT+SM** | - | - | - | - | - | NA | 0.4404 |

|  |  | | |  | | | |
| --- | --- | --- | --- | --- | --- | --- | --- |
| **Zhejiang** | **TST** | **QFT-GIT** | **SM** | **TST+ QFT-GIT** | **TST+QFT-GIT** | **QFT-GIT+SM** |  |
| **TST** | NA | 0.0113 | <0.0001 | 0.0006* | 0.1772 | 0.5102 | **0.0006**** |
| **QFT-GIT** | - | NA | <0.0001 | <0.0001* | <0.0001* | 0.0846 | **<0.0001**** |
| **SM** | - | - | NA | <0.0001* | <0.0001* | **<0.0001*** | **<0.0001**** |
| **TST+QFT-GIT** | - | - | - | NA | 0.0518 | <0.0001* | 1.000 |
| **TST+SM** | - | - | - | - | NA | 0.0298 | 0.0518 |
| **QFT-GIT+SM** | - | - | - | - | - | NA | **<0.0001**** |

**Footnote:** PTB: pulmonary tuberculosis; TST: tuberculin skin test (with a 15 mm cut-off point); QFT-GIT: QuantiFERON® Gold In-Tube (indeterminate results are included as negative results; SM: smear microscopy.

* Significant after Bonferroni correction for 5 multiple comparisons, p value≤0.01; ** significant after Bonferroni correction for 7 multiple comparisons, p value≤0.007.
